# Allele-specific silencing of the gain-of-function mutation in Huntington’s disease using CRISPR/Cas9

**DOI:** 10.1172/jci.insight.141042

**Published:** 2022-10-10

**Authors:** Jun Wan Shin, Eun Pyo Hong, Seri S. Park, Doo Eun Choi, Ihn Sik Seong, Madelynn N. Whittaker, Benjamin P. Kleinstiver, Richard Z. Chen, Jong-Min Lee

**Affiliations:** 1 Center for Genomic Medicine, Massachusetts General Hospital, Boston, Massachusetts, USA.; 2 Department of Neurology, Harvard Medical School, Boston, Massachusetts, USA.; 3 Medical and Population Genetics Program, The Broad Institute of M.I.T. and Harvard, Cambridge, Massachusetts, USA.; 4 Department of Pathology, Massachusetts General Hospital, Boston, Massachusetts, USA.; 5 Department of Pathology, Harvard Medical School, Boston, Massachusetts, USA.; 6 CHDI Foundation, Princeton, New Jersey, USA.

**Keywords:** Genetics, Genetic diseases

## Abstract

Dominant gain-of-function mechanisms in Huntington’s disease (HD) suggest that selective silencing of mutant *HTT* produces robust therapeutic benefits. Here, capitalizing on exonic protospacer adjacent motif–altering (PAM-altering) SNP (PAS), we developed an allele-specific CRISPR/Cas9 strategy to permanently inactivate mutant *HTT* through nonsense-mediated decay (NMD). Comprehensive sequence/haplotype analysis identified SNP-generated NGG PAM sites on exons of common *HTT* haplotypes in HD subjects, revealing a clinically relevant PAS-based mutant-specific CRISPR/Cas9 strategy. Alternative allele of rs363099 (29th exon) eliminates the NGG PAM site on the most frequent normal *HTT* haplotype in HD, permitting mutant*-*specific CRISPR/Cas9 therapeutics in a predicted ~20% of HD subjects with European ancestry. Our rs363099-based CRISPR/Cas9 showed perfect allele specificity and good targeting efficiencies in patient-derived cells. Dramatically reduced mutant *HTT* mRNA and complete loss of mutant protein suggest that our allele-specific CRISPR/Cas9 strategy inactivates mutant *HTT* through NMD. In addition, GUIDE-Seq analysis and subsequent validation experiments support high levels of on-target gene specificity. Our data demonstrate a significant target population, complete mutant specificity, decent targeting efficiency in patient-derived cells, and minimal off-target effects on protein-coding genes, proving the concept of PAS-based allele*-*specific NMD-CRISPR/Cas9 and supporting its therapeutic potential in HD.

## Graphical abstract

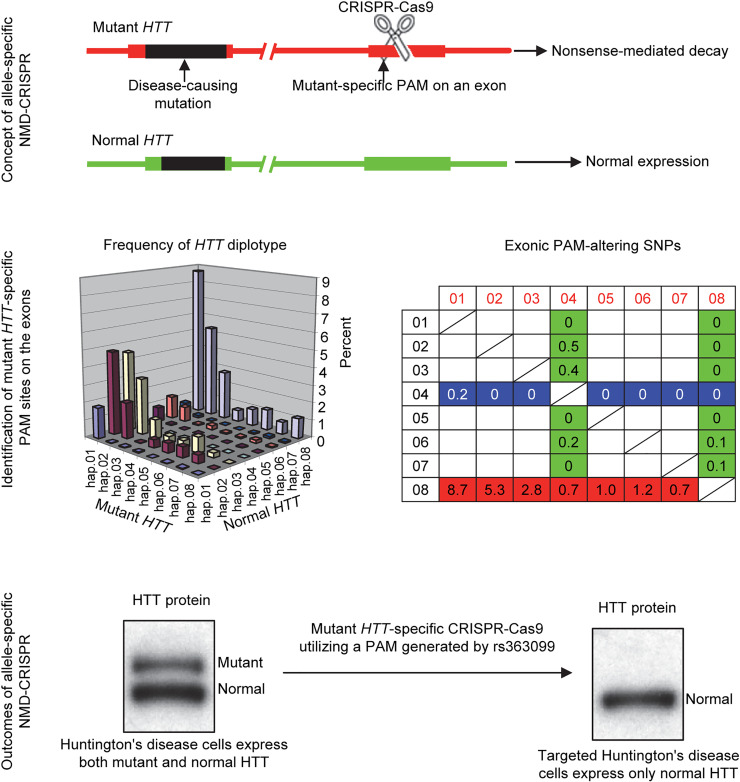

## Introduction

Huntington’s disease (HD) is caused by an expanded CAG trinucleotide repeat in the first exon of the huntingtin gene (*HTT)* (1). CAG repeats greater than 35 generate characteristic motor symptoms in patients whose onset age is inversely correlated with the length of the repeat (2). Overall, the size of the expanded CAG repeat explains approximately 60% of the variance in age at onset in a fully dominant fashion (3), with unexplained variance being associated with various genetic loci (4, 5). These indicate that the rate of HD is primarily determined by the size of the CAG repeat and is further modified by other genes (5). Although the cause of HD has been known for more than 25 years (1), effective treatments have not been developed yet, potentially due to the complicated underlying disease biology in HD.

Considering dominant inheritance in HD (6), the known disease-causing mutation (1), and an essential role for *HTT* in development (7–9), selective inactivation of mutant *HTT* through CRISPR/Cas9 genome editing may yield robust therapeutic benefits. Since an expanded *HTT* CAG repeat causes HD, one may advocate therapeutic strategies that directly target the expanded repeat. However, targeting the disease-causing mutation using CRISPR/Cas9 is challenging and not desirable in HD because of (a) the lack of NGG protospacer adjacent motif (PAM) sequence (which is required for the most commonly used Cas9 endonuclease) in the CAG repeat region, (b) potential inactivation of normal *HTT*, and (c) concomitant targeting of many other genes containing a CAG repeat. Considering that DNA modifications by CRISPR/Cas9 produce permanent changes, the most critical requirement of therapeutic CRISPR/Cas9 strategies for HD is allele specificity. To inactivate only mutant *HTT* without targeting the disease-causing mutation, we developed an allele-specific CRISPR/Cas9 strategy that selectively targets the *HTT* haplotype harboring an expanded CAG repeat using PAM sites generated by SNPs (10). Using the PAM-altering SNP–based (PAS-based) dual guide RNA (gRNA) CRISPR/Cas9 approach, we were able to selectively excise a genomic region including the transcription start site and an expanded CAG repeat from the mutant *HTT* locus, allowing cells to produce only normal *HTT* mRNA and protein (10). Our PAS-based haplotype-targeting CRISPR/Cas9 strategy using 2 gRNAs to prevent the transcription of the mutation-bearing transcript represents a highly flexible approach that can be applied to a gene of interest regardless of the location, size, and type of the disease-causing mutation (10). However, the use of 2 gRNAs required for genomic excision to prevent the transcription of mutant *HTT* can increase the possibility of off-targeting. In this study, we developed a complementary CRISPR/Cas9 strategy that uses a single gRNA to selectively inactivate the mutant *HTT* through nonsense-mediated decay (NMD) (11, 12), and we subsequently determined its applicability, allele-specificity, molecular consequences, and off-target effects to evaluate its utility in clinical applications.

## Results

### PAS-based mutant-specific NMD-CRISPR/Cas9 strategies for HD.

**Figure 1:**
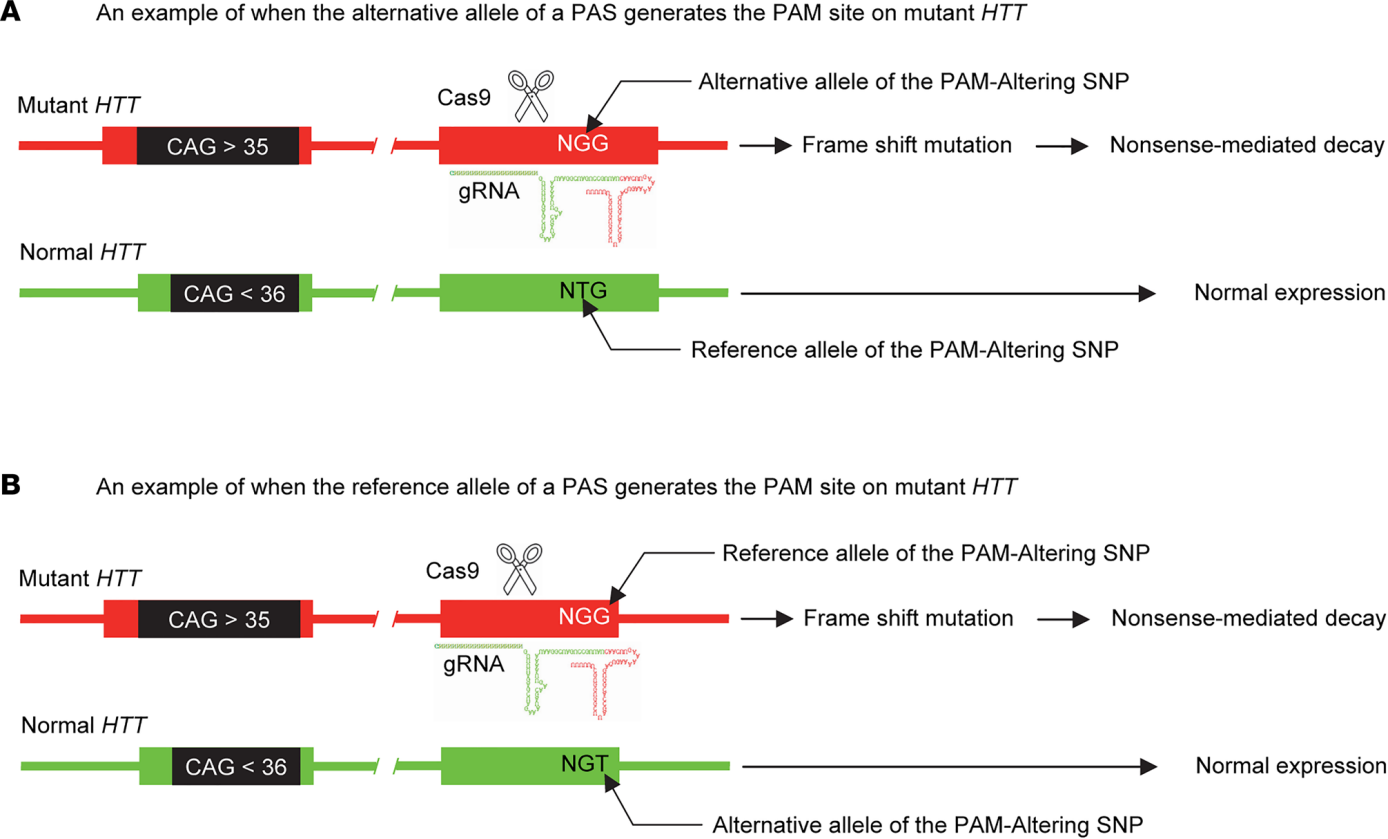
The concept of allele-specific NMD-CRISPR/Cas9 strategy capitalizing on exonic PAM-altering SNP. The concept of allele-specific CRISPR/Cas9 utilizing an exonic PAM-Altering SNP (PAS) is illustrated. (**A** and **B**) Alternative alleles of certain SNPs generate (**A**) and eliminate (**B**) CRISPR/Cas9 PAM sites (i.e., NGG for *Sp*Cas9). A CRISPR/Cas9 strategy using an exonic NGG PAM site that exists only on the mutant *HTT* (red in **A** and **B**) is predicted to induce nonsense-mediated decay of mutant *HTT* mRNA selectively without impacting the expression of the normal *HTT* (green).

**Figure 2:**
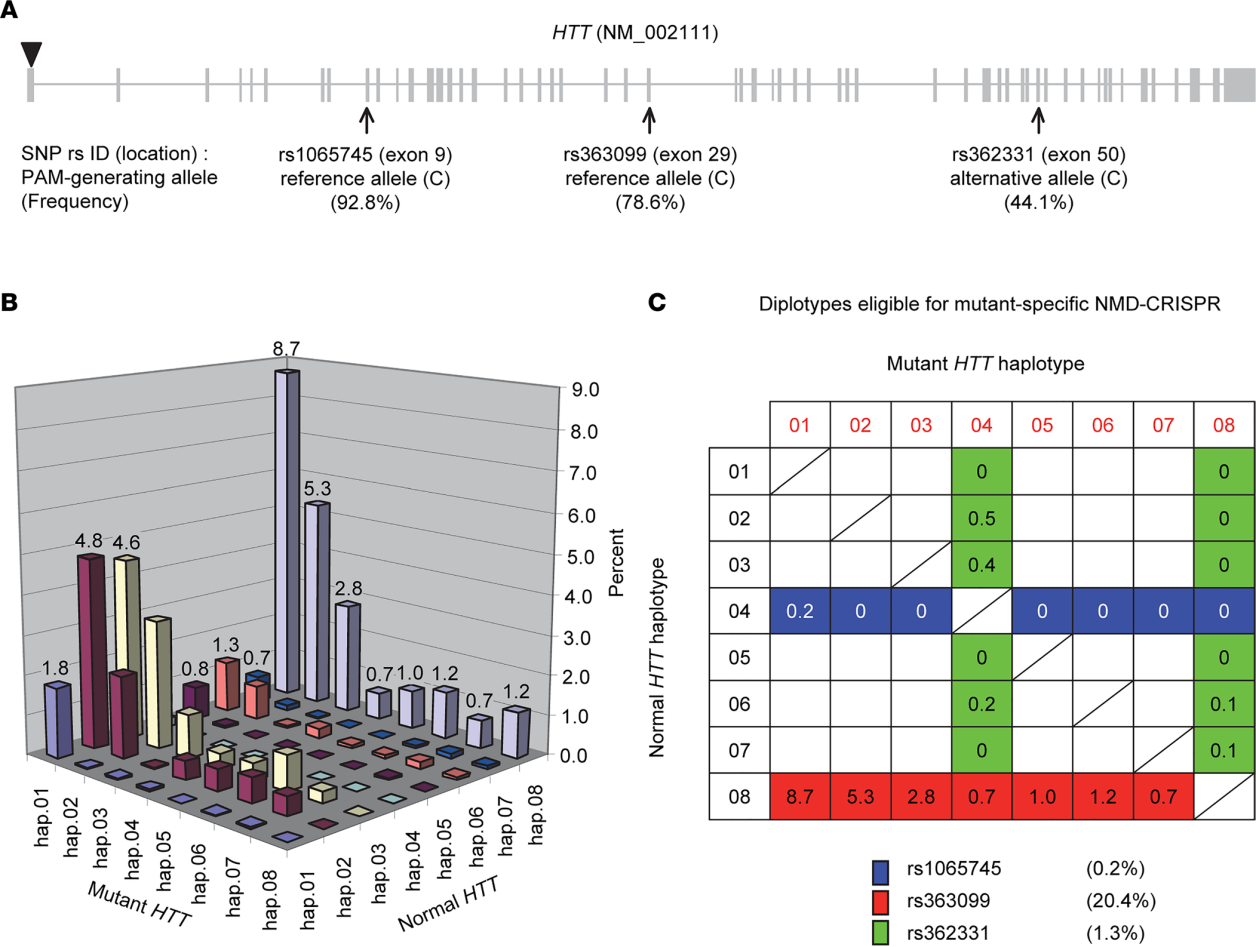
PAM-altering SNPs (PAS) that permit mutant *HTT*-specific NMD-CRISPR/Cas9 in HD. (**A**) In order to identify PAM sites that allow mutant-specific CRISPR/Cas9 in HD, PAS on *HTT* coding exons were identified from KGP data. Subsequently, PAS-generated NGG PAM sites were mapped to the 8 common *HTT* haplotypes. Among 91 exonic PAS on *HTT*, alleles of 3 exonic PAS (arrows) are polymorphic in the 8 common *HTT* haplotypes, permitting mutant-specific CRISPR/Cas9 in European HD subjects with common diplotypes. (**B**) To estimate the levels of mutant specificity of those 3 polymorphic PAS, we calculated the proportion of each diplotype in HD subjects with European ancestry, focusing on the 8 common haplotypes. Percentage values for the most frequent haplotypes in the disease and normal chromosomes (hap.01 and hap.08) are provided. (**C**) We identified *HTT* diplotypes carrying mutant-specific PAM sites generated by those 3 polymorphic PAS and calculated the levels of mutant specificities. Table cells in blue, red, and green represent the NGG PAM site on the mutant *HTT* generated by rs1065745, rs363099, and rs362331, respectively.

Among other requirements, mutant *HTT* specificity is strongly preferred for DNA-targeting therapeutic strategies for HD, as they produce permanent changes. Our complementary allele-selective CRISPR/Cas9 strategy for HD aims at inducing NMD of mutant *HTT* mRNA (namely NMD-CRISPR/Cas9) by targeting a mutant *HTT*-specific exonic PAM site that is produced by the alternative allele (Figure 1A) or the reference allele of a given PAS (Figure 1B). All cases of HD are due to an expanded CAG repeat. However, the disease-causing mutation is found on diverse haplotypes (13–15), and many HD patients carry different combinations of mutant and normal *HTT* haplotypes (i.e., diplotype) (14, 16). Therefore, key steps in developing mutant-specific NMD-CRISPR/Cas9 strategies for HD are: (a) finding exonic PAS on *HTT*, (b) mapping exonic PAS-generated PAM sites on *HTT* haplotypes, and (c) identifying mutant *HTT*-specific PAM sites in a given diplotype. Aiming at identifying exonic PAS, we analyzed 1,000 Genomes Project (KGP) data and revealed that 91 coding SNPs on *HTT* alter the PAM sequence for *Sp*Cas9 (*S*. *pyogenes* Cas9; 5′-NGG-3′) (Supplemental Table 1 and Supplemental Figure 1; supplemental material available online with this article; https://doi.org/10.1172/jci.insight.141042DS1). We then mapped 91 PAS-generated NGG PAM sites on the 8 most frequent *HTT* haplotypes, which account for more than 80% of HD subjects with European ancestry (Supplemental Figure 2). Among 91 PAS, 69 and 19 SNPs generate NGG PAM sites on all and none of the 8 common *HTT* haplotypes, respectively. Therefore, those SNP variants can’t be used for allele-specific CRISPR/Cas9 for HD subjects carrying common diplotypes (Supplemental Figure 2). However, 3 exonic PAS generate PAM sites on some of the common *HTT* haplotypes (Figure 2A). For example, reference alleles of rs1065745 and rs363099 generate NGG PAM sites on hap.04 and hap.08, respectively. In contrast, the alternative allele of rs362331 generates NGG PAM sites on hap.04 and hap.08 haplotypes (Supplemental Figure 2).

Having identified candidate variants that might permit mutant *HTT*-specific NMD-CRISPR/Cas9 in HD, we then evaluated the levels of mutant specificity of those SNPs by calculating the percentages of HD subjects who carry the PAM site only on the mutant *HTT*. Firstly, we calculated the proportion of each diplotype in HD subjects based on our large-scale genome-wide association study data that were analyzed to identify genetic modifiers (4). In agreement with previous studies with small sample sizes (14, 17), hap.01 and hap.08 are the most frequent mutant and normal haplotypes, respectively. As a result, a diplotype comprising mutant *HTT* on hap.01 and normal *HTT* on hap.08 represents the most frequent diplotype in HD, accounting for more than 8% of HD subjects with European ancestry (Figure 2B). Focusing on rs1065745, rs363099, and rs362331, we then identified diplotypes that carry NGG PAM sites selectively on the mutant *HTT*. HD subjects with normal *HTT* on hap.04 and mutant *HTT* on non-hap.04 carry an rs1065745-generated PAM sequence on the mutant chromosome, accounting for approximately 0.2% of European HD subjects with common diplotypes (Figure 2C, blue). HD subjects carrying mutant *HTT* on hap.04 or hap.08 and normal *HTT* on other haplotypes have an rs362331-generated NGG PAM site on the disease chromosome, accounting for approximately 1.3% of HD (Figure 2C, green). In contrast to low levels of mutant specificity for rs1065745 and rs362331, rs363099 showed a significantly higher mutant specificity. The alternative allele of rs363099 eliminates the NGG PAM site on the most common normal *HTT* haplotype (i.e., hap.08); therefore, HD subjects with mutant *HTT* on non-hap.08 and normal *HTT* on hap.08 carry a NGG PAM site only on the mutant *HTT* (Figure 2C, red), accounting for approximately 20% of European HD subjects with common diplotypes (Figure 2B). When considering 16 common *HTT* haplotypes, which account for more than 90% of the mutant chromosomes (14), approximately 21.5% of HD subjects have an NGG PAM selectively on the mutant *HTT* at rs363099 because hap.08 is still the only haplotype that does not carry an NGG PAM at this location. Last, 23.1% of HD subjects in our genome-wide association study (18) are heterozygous at rs363099 and carry a normal hap.08 haplotype, suggesting that approximately 23% of HD subjects are eligible for a mutant-specific CRISPR/Cas9 strategy utilizing the PAM site generated by rs363099. When directly based on the phased alleles at rs363099, 28.5% and 2.8% of HD subjects carry the NGG PAM site selectively on mutant *HTT* and selectively on normal *HTT*, respectively, revealing relatively similar mutant specificity at this locus.

### Allele specificity of NMD-CRISPR/Cas9 based on rs363099.

**Figure 3:**
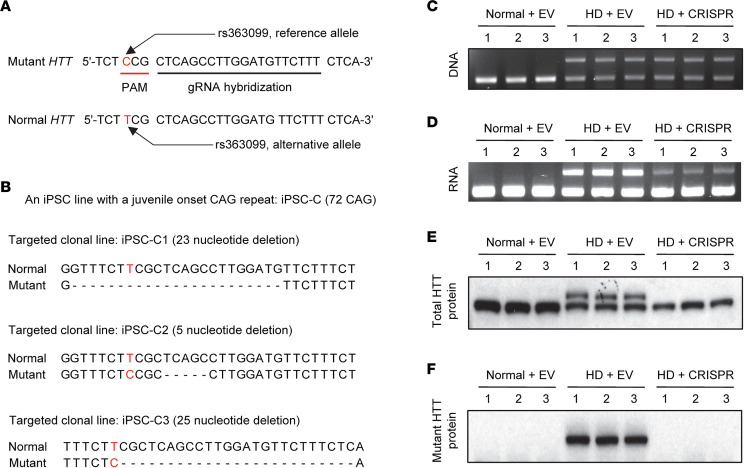
Molecular consequences of mutant *HTT-*specific CRISPR/Cas9. (**A**) Our mutant-specific CRISPR/Cas9 strategy using the NGG PAM site generated by rs363099 is illustrated. The NGG PAM site occurs on the minus strand of the mutant *HTT* (red underline). The PAS (rs363099, red) and the crRNA hybridization site (black underline) are highlighted. (**B**) An HD iPSC with a juvenile-onset CAG repeat was transfected with a plasmid vector expressing either *Sp*Cas9 only (empty vector [EV]) or *Sp*Cas9/gRNA to establish targeted clonal lines. Three non-HD single-cell clones were also developed after EV treatment. Three independent targeted single-cell clones with inactivated mutant *HTT* were confirmed by Sanger sequencing. All 3 clonal lines showed out-of-frame deletion at the 29th exon. Dashed lines represent deletions. (**C**–**F**) Subsequently, 3 independent clones for non-HD control (left; Normal + EV), EV-treated HD clones (middle; HD + EV), and CRISPR/Cas9 treated HD clones (right; HD + CRISPR) were analyzed to characterize *HTT* DNA, RNA, and protein (*n* = 3 for each group). (**C**) The presence of an expanded CAG repeat in the DNA of the targeted clones (upper band) was confirmed by PCR assay of genomic DNA. (**D**) The expression levels of *HTT* mRNA were measured by RT-PCR analysis designed to detect the CAG repeat region. (**E** and **F**) Total and mutant HTT protein levels were determined by immunoblot analysis using a panhuntingtin antibody (MAB2166) and mutant huntingtin-specific antibody (1F8), respectively. Images of the full gels of immunoblot analysis are provided in Supplemental Figure 7.

Previous studies have demonstrated complete allele specificities and robust molecular outcomes of PAS-based dual gRNA CRISPR/Cas9 approaches to prevent the transcription of the mutant *HTT* (10, 19). In addition, preclinical efficacy of permanent suppression of mutant *HTT* expression in a knock-in mouse model (20) and the highest mutant specificity of rs363099 in HD subjects (this study) make a CRISPR/Cas9 strategy based on this variant relevant and significant. Therefore, we set out to determine the editing efficiency, allele specificity, and molecular consequences of a single gRNA CRISPR/Cas9 strategy utilizing the PAM site generated by rs363099. We reasoned that the levels of allele specificity would be independent of cell types because DNA sequence provides the basis for allele specificity in our CRISPR/Cas9 strategy. Therefore, we used readily available induced pluripotent stem cell (iPSC) lines derived from HD patients carrying mutant hap.01 and normal hap.08 as a representative diplotype for subsequent molecular analyses focusing on evaluating the levels of allele specificity. As summarized in Figure 3A, our allele-specific CRISPR/Cas9 strategy was designed to selectively target the mutant *HTT* haplotype using the NGG PAM site on the minus strand of the disease chromosome to induce NMD of mutant *HTT* mRNA. The lack of a PAM site at the same location on the normal chromosome is predicted to prevent the CRISPR/Cas9 genome editing in normal allele, leaving normal *HTT* expression intact (Figure 3A). Indeed, our rs363099-based CRISPR/Cas9 strategy showed a perfect allele specificity in iPSC lines derived from heterozygous HD subjects with adult-onset CAG repeats, generating small indels selectively on the mutant *HTT* without modifying its normal counterpart (Table 1). For example, iPSC-A carrying 46 CAGs showed that 16.07% of mutant alleles and 0% of normal alleles were edited by transfection of Cas9 and our test gRNA without puromycin selection (Table 1 and Supplemental Table 2). Similarly, transfection experiments on an independent iPSC line carrying 42 CAGs (iPSC-B) showed that ~23% of mutant alleles were edited without targeting normal *HTT* (Table 1 and Supplemental Table 2). Consistent with a previous report (21), our CRISPR/Cas9 strategy produced out-of-frame indels predominantly as zero, and a small number of in-frame indels were observed in the mutant alleles of iPSC-A and iPSC-B, respectively (Table 1 and Supplemental Table 2). We do not think the difference in the levels of in-frame modification between iPSC-A and iPSC-B was due to the difference in CAG sizes. Rather, this might be due to higher editing efficiencies in iPSC-B, generating more diverse genome modifications at the target site. Complete allele specificity, good targeting efficiencies, and significant reduction in total HTT protein levels were also observed in HD patient–derived neural precursor cells (NPCs) (Supplemental Figure 3) and other cell types (data not shown). Cell type–independent allele specificity is quite expected because DNA sequence provides the basis for allele discrimination in our strategy. In contrast to our primary allele-specific targeting strategy based on a PAS, we observed inactivation of normal *HTT* when rs363099 was targeted as part of gRNA hybridization (Supplemental Figure 4), supporting a better allele specificity for a PAS-based CRISPR/Cas9 approach at this location. Together, high levels of mutant specificity and decent editing efficiency of our rs363099-based NMD-CRISPR/Cas9 strategy observed in our transfection experiments (without selection) suggest that single SNP-based haplotype-targeting CRISPR/Cas9 approaches can selectively inactivate the disease allele without directly targeting the mutation itself.

### Complete ablation of mutant HTT protein expression by allele-specific NMD-CRISPR/Cas9.

Next, we developed targeted clonal lines to unequivocally determine the immediate downstream consequences of our rs363099-based mutant-specific NMD-CRISPR/Cas9. We chose an iPSC with a juvenile-onset CAG repeat (72 CAGs) in order to distinguish mutant HTT protein from its normal counterpart in immunoblot assays. Three independent clonal cell lines were established (Figure 3B); subsequent sequencing analysis confirmed premature early stop codons at 29th, 30th, and 29th exons in iPSC-C1, iPSC-C2, and iPSC-C3 lines, respectively, predicting selective NMD of mutant *HTT* mRNA. Consistent with our predictions, targeted HD single-cell clones showed both expanded and normal CAG repeats in DNA (Figure 3C). However, mutant *HTT* mRNA was significantly reduced (Figure 3D, Supplemental Figure 5, and Supplemental Figure 6A), and mutant HTT protein was completely ablated (Figure 3, E and F; Supplemental Figure 6B; and Supplemental Figure 7). Complete loss of mutant HTT protein and the lack of fragmented mutant HTT protein in targeted clonal lines (Supplemental Figure 8) suggest that (a) mutant *HTT* mRNA is degraded quickly before producing full-length or fragmented mutant HTT protein in targeted cells, and (b) the small amount of mutant *HTT* mRNA detected by reverse transcription PCR (RT-PCR) assays (Figure 3D) and MiSeq analysis of cDNA (Supplemental Figure 5) represent newly synthesized mutant *HTT* mRNA that has not been subjected to NMD yet.

### Molecular consequences of mutant HTT-specific NMD-CRISPR/Cas9.

**Figure 4:**
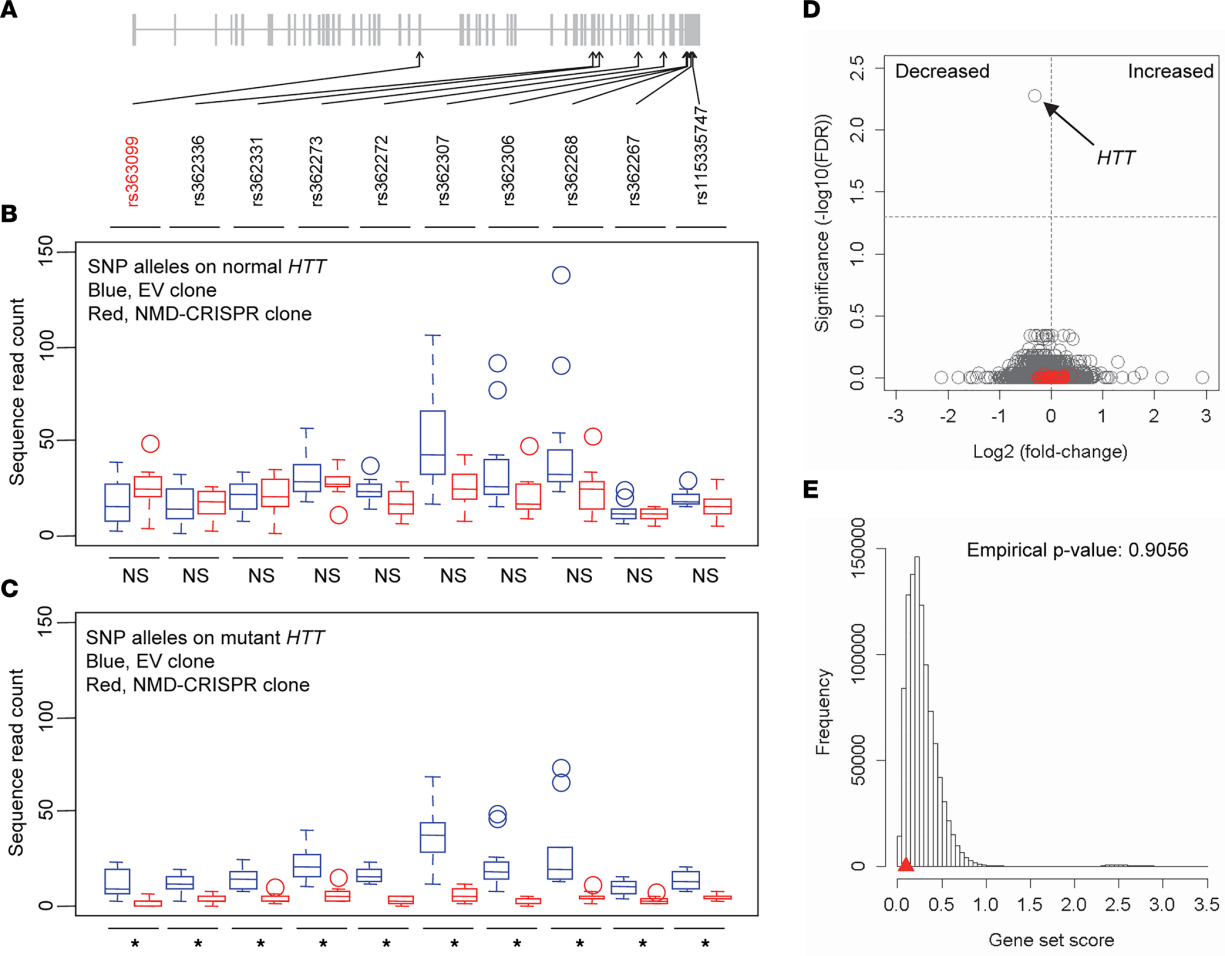
Allele specificity and on-target gene selectivity of mutant *HTT*-specific CRISPR/Cas9. (**A**) Two iPSC lines (iPSC-A and iPSC-B) carrying expanded CAG repeats on hap.01 (mutant *HTT*) and normal CAG repeats on hap.08 haplotype (normal *HTT*) were analyzed by RNA-Seq. This most frequent diplotype carries 10 heterozygous SNPs on exons of *HTT* (including our target PAS rs363099; red), permitting ASE analysis of RNA-Seq data. (**B**) For each SNP site, we counted the RNA-Seq sequence reads carrying hap.08 alleles (normal *HTT)* in EV-treated clonal lines (blue; *n* = 12) and those in CRISPR/Cas9-treated clonal lines (red; *n* = 12 samples/group). Each box plot shows the maximum, upper quarter, median, lower quarter, and minimum based on *n* = 12 independent clones/group. Circles represent outliers defined by a standard interquartile outlier detection method. Student *t* test (2 tailed) was performed separately to determine the statistical significance of each site. None of them were significant by a Bonferroni multiple-test–corrected *P* value. (**C**) Similarly, we compared sequence read counts of alleles of hap.01 at 10 heterozygous exonic SNPs (representing mutant *HTT*) in EV- and CRISPR/Cas9-treated clones (*n* = 12 samples/group) and performed Student *t* test (2 tailed). Asterisks denote statistical significance after Bonferroni multiple-test correction (corrected *P* < 0.05). (**D**) DGE analysis was performed to identify genes whose expression levels were altered by our mutant-specific CRISPR/Cas9. A volcano plot was based on 16,840 expressed genes in our clonal lines. The *y* axis and *x* axis represent the levels of significance (–log_10_[FDR]) and effect size (log_2_[fold change]), respectively. A dashed horizontal line represents the significance threshold (FDR = 0.05). Red circles represent genes containing or flanking the predicted off-target sites. (**E**) We also tested whether a gene set comprising all predicted off-targets was significantly enriched by comparing the true gene set score (sum of significance values) of predicted off-targets (red triangle) to a null distribution of gene set scores obtained from 1,000,000 random sampling of 52 genes.

Having confirmed high levels of allele specificity, we characterized the molecular outcomes of our mutant-specific CRISPR/Cas9 strategy using 2 iPSC lines carrying adult-onset CAG repeats (iPSC-A and iPSC-B carrying 42 and 46 CAGs, respectively). We performed RNA-Seq analysis of targeted single-cell clones in order to minimize noise. Multiple independent clonal lines for experimental (Supplemental Table 3; *n* = 12) and control group (Supplemental Table 4; *n* = 12) were developed using our rs363099-based CRISPR/Cas9 strategy. Out-of-frame indels in targeted clones were confirmed by Sanger sequencing and MiSeq analysis of genomic DNA, and they were further validated by MiSeq analysis of cDNA (Supplemental Table 3). Similar to targeted clonal lines with a juvenile-onset CAG repeat (Figure 3D and Supplemental Figure 5), we detected low levels of mutant *HTT* mRNA (Supplemental Table 3) in the absence of mutant protein (data not shown) in our targeted clones for RNA-Seq analysis. These data suggest that targeted clonal lines continuously produce both mutant and normal *HTT* mRNA, but most of the mutant *HTT* mRNA is quickly degraded before producing mutant HTT protein. We then performed RNA-Seq analysis to determine the molecular consequences of our mutant *HTT*-specific CRISPR/Cas9 strategy based on rs363099. Firstly, allele-specific expression (ASE) analysis of *HTT* focusing on sequence reads containing heterozygous coding SNPs (10 SNPs for hap.01/hap.08 diplotype, including rs363099; Figure 4A) did not reveal any significant differences between empty vector (EV) controls and targeted clones regarding alleles on normal *HTT* (Figure 4B and Supplemental Figure 9A). However, all 10 heterozygous SNP sites showed significantly (multiple-test–corrected *P* < 0.05) decreased mutant *HTT* levels in targeted clones (Figure 4C and Supplemental Figure 9B). Second, we performed genome-wide differential gene expression (DGE) analysis to identify genes significantly altered in targeted HD clones compared with EV-treated HD cells. Interestingly, the shape of the volcano plot was atypical (Figure 4D), rather resembling that of random sample comparisons (Supplemental Figure 10). These findings indicate that expression levels of genes in targeted clones were mostly unchanged except for *HTT* (Figure 4D). Since *HTT* was the only significantly altered gene in targeted clonal lines (Figure 4D, black arrow), these data also imply that the probability of recurring frameshift mutations that can alter gene expression of other protein-coding genes may be low in our CRISPR/Cas9 strategy utilizing the PAM site generated by rs363099.

### Analysis of genome-wide off-target effects.

Last, we evaluated the off-target effects of our rs363099-based mutant *HTT*-specific CRISPR/Cas9 strategy. We performed the genome-wide unbiased identification of double-strand breaks enabled by sequencing (GUIDE-seq) assay to identify potential off-target sites. To increase the sensitivity of off-target site detection, we used HEK293T cells, which generally exhibit high transfection efficiency and robust CRISPR/Cas9 editing. Transfection of gRNA for our NMD-CRISPR/Cas9 strategy and Cas9 without puromycin selection resulted in approximately 60%–70% on-target editing efficiency in HEK293T cells, and subsequent GUIDE-seq analysis revealed 6 potential off-target sites (Supplemental Figure 11A). Three sites located in the intergenic regions (off-target #1, #2, and #3) showed modest levels of modification compared with the levels of on-target editing in HEK293T cells (Supplemental Figure 11A). The other 3 potential off-targets located in an intron (off-target #4) or intergenic regions (off-target #5 and #6) showed low levels of genome editing in HEK293T cells (Supplemental Figure 11A). To validate those 6 potential off-target sites in HD cells, we analyzed patient-derived iPSC lines treated with EV or our test gRNA. MiSeq analysis of representative HD iPSC samples showed relatively modest genome editing at off-target sites #1, #2, and #3 (1%–6%) and no modification at sites #4, #5, or #6 (Supplemental Figure 11B). Although off-target editing was detected at 3 sites in the patient-derived cells, off-target genome editing at those sites may not result in functional changes because of their locations relative to genes. Our RNA-Seq data also show that the expression levels of genes flanked by or harboring potential off-target sites were not altered in our targeted clones (Supplemental Table 5), suggesting that (a) targeted clonal lines were not edited at off-target sites repeatedly and/or (b) genome editing at the 6 potential off-target sites does not alter the expression of protein-coding genes.

To further evaluate off-target effects, we used an in silico off-target prediction algorithm (Cas-OFFinder). A total of 83 sites were predicted for our primary test gRNA based on rs363099 (Supplemental Table 6); none of the predicted sites had a perfect match. The site with the highest prediction score contains 1 mismatch (chr12: 92165291–92165313; located at an intergenic region), but we did not detect any modification at this location in iPSC-C cells (Supplemental Table 7). Moreover, only 1 predicted off-target site is located in an exon (*PLXNA1*; containing 2 mismatches and 1 bulge) (Supplemental Table 6). The predicted off-target sites were mapped to or are flanked by 76 unique genes. Among them, 53 genes were expressed in our control HD iPSC clones, permitting evaluation of expression levels of predicted off-targets in our RNA-Seq data. Notably, genes harboring or located near the predicted off-target sites were neither significantly altered individually (Figure 4D, red circles) nor enriched as a group in our RNA-Seq data (Figure 4E). These data indicate that our PAS-based CRISPR/Cas9 strategy utilizing rs363099 is unlikely to alter expression levels of protein-coding genes harboring or flanking off-target sites identified by GUIDE-seq or predicted by a prediction algorithm.

## Discussion

Causative mutations of Mendelian disorders are highly sought after because subsequent revelation of underlying disease mechanisms has been thought to lead to cures. However, detailed mechanistic studies rarely have produced effective therapeutics. For example, in several dominant disorders (e.g., some forms of amyotrophic lateral sclerosis, Alzheimer’s disease, and HD), the disease-producing genetic defects have been known for more than 20 years (22–25), but no effective intervention has yet been developed. This suggests that defining drug targets in genetic diseases through mechanism-focused studies is challenging. Nevertheless, with the evolution of various gene-targeting approaches, the disease-causing gene itself is recognized as the best therapeutic target, even without a full understanding of its biological functions.

Various gene targeting technologies have been developed. Gene-knockdown or -KO approaches can be broadly grouped based on the target. RNA interference (RNAi) and antisense oligonucleotide (ASO) interact with RNA to produce reversible knockdown of the target, offering a versatile means of gene targeting. However, these approaches may show high levels of off-target effects (26–30) and require repeated treatments (31–33). In contrast, zinc finger and CRISPR/Cas approaches aim at producing changes in the target DNA to generate irreversible KO effects. Although they provide overall high levels of on-target gene specificity, delivery to target tissue is a major challenge to overcome to apply these powerful tools in humans (26). Technologies for lowering the levels of mRNA have yielded some successes in model systems of HD (34–39). Furthermore, a phase 1-2a trial showed dose-dependent reduction of mutant HTT protein by non–allele-specific ASO in humans (40), supporting the feasibility of gene-knockdown approaches. Although promising, non–allele-specific RNA-lowering approaches have limitations including difficulty in adequately maintaining the levels of mutant *HTT* mRNA. Importantly, despite therapeutic efficacies of non–allele-specific HTT-lowering approaches in preclinical studies, a phase III trial to test a non–allele-specific ASO in HD showed the lack of clinical benefits (41, 42). Considering these, alternative allele-specific DNA-targeting approaches may produce robust therapeutic benefits (31, 43–47) because they may overcome limitations of non–allele-specific RNA-targeting approaches (26).

Targeted disruption of *Htt* causes embryo lethality (7–9) and other deficits in mice (48–50). Also, insufficient *HTT* levels due to compound heterozygous mutations are associated with developmental problems in humans (51, 52). However, 1 copy of *Htt* (i.e., heterozygous KO) is sufficient to support the survival of mice (7, 9), and individuals with 1 functional copy of *HTT* do not present HD symptoms or developmental problems (51–53). Given that HD is caused by a dominant gain-of-function mutation (1, 54), these observations suggest that selective inactivation of the mutant *HTT* gene may produce significant clinical benefits without side effects. Considering genome engineering produces permanent changes, high levels of allele specificity are strongly preferred for any therapeutic DNA-targeting strategy for HD. We, thus, focused on developing allele-specific CRISPR/Cas9 strategies capitalizing on genetic variations that generate or eliminate a PAM site. We conceived 2 PAS-based allele-specific CRISPR/Cas9 strategies to selectively inactivate the mutant *HTT* in a given HD subject. Previously, using 2 gRNAs, we simultaneously targeted 2 mutant-specific PAM sites that encompass the transcription start site and an expanded CAG repeat of the mutant *HTT* to prevent the transcription of the mutant allele by genomic deletion (namely, Transcription Prevention–CRISPR/Cas9) (10). Similar approaches have been tested in patient-derived fibroblasts and mouse models of HD (19, 20). As a complementary approach, this study tested the concept of allele-specific CRISPR/Cas9 targeting an exonic PAM site present only on the mutant *HTT* in a given HD subject to induce NMD of the mutant *HTT* mRNA. Based on targeted patient-derived cells, both strategies resulted in complete ablation of mutant HTT protein without impacting the expression of the normal counterpart, demonstrating high levels of allele selectivity of PAS-based approaches. Also, the timing of treatment is an important subject for progressive neurodegenerative disorders like HD. An essential role for *HTT* in development (7, 8, 49, 50) may argue against applications of gene-targeting treatments in young HD subjects. By contrast, sufficient clinical efficacy may not be achieved if patients are treated after clinical manifestation, when significant neurodegeneration has already occurred (55). These observations support the value of our allele-specific DNA-targeting strategy as the means for early treatment because it may be applied to presymptomatic mutation carriers without producing significant adverse effects.

Since the difference in DNA sequence between mutant and normal *HTT* in a given HD subject serves as the basis for our personalized mutant-specific NMD-CRISPR/Cas9 strategy, the proportion of HD subjects who are eligible for a CRISPR/Cas9 strategy based on a particular PAS is determined by the frequency of the PAM-generating allele of the target variant on the mutant *HTT*. This study tested the concept of mutant-specific NMD-CRISPR/Cas9 based on a PAS rs363099, and this concept can be applied to approximately 20% of HD subjects with European ancestry. In contrast, a CRISPR/Cas9 strategy directly targeting the CAG repeat may be appealing because it can be applied to 100% of HD subjects. However, such a strategy is technically challenging and may generate adverse outcomes due to (a) the lack of a robust PAM site for the commonly used *Sp*Cas9, (b) decreased allele selectivity, and (c) lower on-target gene specificity. Although not applicable to all individuals with the target disease, personalized medicine is an important and relevant direction of health care because it may provide increased safety and efficacy on an individual level. In light of this view, our PAS-based CRISPR/Cas9 represents one of the personalized HD therapeutic strategies that permit high-level efficacy and safety owing to high levels of mutant *HTT* specificity. We reason that identification of DNA variants that are compatible with diverse engineered Cas9 variants with different PAM specificities (56–62) will significantly increase the applicability of PAS-based mutant-specific NMD-CRISPR/Cas strategies for HD.

The identification of a PAS (i.e., rs363099) that has never been targeted in HD by CRISPR/Cas9 represents a potentially novel discovery. In addition, our data demonstrating good targeting efficiency, high levels of allele-specificity, and minimal impacts on other protein-coding genes in patient-derived iPSC lines support the clinical relevance of the mutant-specific CRISPR/Cas9 strategy using rs363099. We also evaluated the feasibility of our allele-specific CRISPR/Cas9 strategy in neurons using differentiated neuronal cells from iPSC-B (42 CAG) as a model system. As predicted from the low transduction efficiencies of adeno-associated viruses (AAVs) in differentiated neurons from iPSC (63), transduction of AAV6, AAV8, AAV9, or AAV PHP.eB did not produce modification at the target site in our iPSC-derived neurons. The lack of CRISPR/Cas9 editing by AAV serotypes that we tested in neurons might be due to low transduction efficiency (not low genome editing efficiency) because of (a) the proof of CRISPR/Cas9 in differentiated iPSC-derived neurons using an inducible system (64, 65) and (b) successful CRISPR/Cas9 genome editing in postmitotic neurons (66–69). In support, efficient CRISPR/Cas9 editing in iPSC-derived neurons is rarely found in the literature, and overall AAV transduction efficiencies were significantly lower in iPSC-cortical neurons compared with other cell types (63). Nevertheless, delivery of ribonucleoprotein (RNP) complex (Cas9 protein and gRNA) and mRNA (Cas9 mRNA and gRNA) yielded modest but complete allele-specific modifications on the mutant *HTT* in differentiated neurons from iPSC-B (Supplemental Figure 12). Considering the clinical relevance and utilities of patient-derived iPSCs and differentiated neurons, development of AAV serotypes that efficiently deliver CRISPR/Cas9 to these cell types will significantly facilitate subsequent optimizations required for the clinical trials.

Due to the lack of reproducible and relevant phenotypes in HD patient-derived iPSCs (70, 71) and iPSC-derived neurons, functional assessments of our rs363099-based mutant-specific NMD-CRISPR/Cas9 strategy in these cell types were impractical. For example, differences in neuronal induction (72–74), levels of nestin (75, 76), action potential (71, 72, 77), HTT protein aggregates (71, 72, 78), and CAG-repeat instability (71, 72, 79, 80) in HD neurons were inconsistent. Also, it was not feasible to determine targeting efficiencies and impacts on behavioral phenotypes of our strategy in vivo because mouse models that specifically permit the evaluation of our rs363099-based NMD-CRISPR/Cas9 do not exist. The Hu97/18 mouse model is heterozygous at rs363099 (81) and, therefore, appears to be suitable for testing our mutant-specific NMD-CRISPR/Cas9 strategy. However, the transgene in Hu97/19 comprises approximately 5 tandem copies of mutant *HTT* (https://www.jax.org/strain/008197); therefore, application of our single-guide RNA–mediated CRISPR/Cas9 strategy is expected to produce both small indels and large genomic deletions because CRISPR/Cas9-mediated large genomic deletions are efficient and frequent (10, 82, 83). Considering potentially unexpected genomic excision by single gRNA, it will be technically challenging to meaningfully interpret the results of NMD-CRISPR/Cas9 experiments using Hu97/18 mice. For example, 20% of editing of mutant allele in Hu97/18 mice by our NMD-CRISPR/Cas9 strategy can be (a) targeting of all 5 copies of mutant *HTT* in 20% of cells, (b) targeting 1 copy of the transgene in all cells, or (c) other combinations. Therefore, developing new HD mouse models carrying only 1 copy of mutant *Htt* with relevant human genetic variations will be critical in precisely determining editing efficiencies and functional consequences of mutant-specific NMD-CRISPR/Cas9 strategies. Since DNA sequence is the basis for the allele specificity of our CRISPR/Cas9 strategy, we expect high levels of allele specificity, regardless of cell type or delivery method. Still, such new mouse models will play an important role in evaluating and optimizing gRNAs and delivery methods, significantly facilitating the development of mutant-specific CRISPR/Cas9 strategies for HD subjects. Taken together, our data showing complete ablation of mutant HTT protein expression and preclinical efficacy of CRISPR/Cas9 strategies to prevent the transcription of mutant *HTT* (20) supports the therapeutic potential of our single gRNA-mediated mutant-specific CRISPR/Cas9 based on rs363099.

Although a non–allele-specific *HTT-*lowering ASO did not generate significant benefits in the first phase III trial, strengths and advantages of such approaches remain important. Identification of improved targets and optimized clinical trial designs may prove the clinical efficacies of non–allele-specific lowering approaches in HD. Alternatively, numerous PAS on the human genome permit widespread application of allele-specific CRISPR/Cas9 (84). Our PAS-based haplotype-targeting approach will be especially powerful when dealing with single gene disorders caused by dominant gain-of-function mutations because it can inactivate the disease-causing mutation regardless of its location, type, or size (10). Areas of CRISPR/Cas9 application are broadening beyond labs quickly. In our study focused on developing allele-specific DNA-targeting strategies using patient-derived iPSC as a model system, the levels of gene editing were somewhat modest. In support, challenges in genome editing in iPSC have been observed in the field (85–87). Despite modest editing in iPSC models, allele-specific CRISPR/Cas9 strategies may generate significant clinical benefits when combined with efficient delivery methods, as supported by robust genome editing in postmitotic neurons (66–68) and significant benefits in mouse models of HD (20, 88). Of note, our data showing on-target gene specificity, mutant allele selectivity, and robust outcomes on the mutant allele addressed critical requirements for safe and effective CRISPR/Cas9 therapeutics. Still, our study was not able to determine the impacts of an allele-specific NMD-CRISPR/Cas9 strategy in mice due to the lack of appropriate preclinical models for HD with correct genomic context, representing major limitations and weaknesses of our current data set. In order to advance our alternative therapeutic strategy, it will be critically important to demonstrate efficacy and safety in the appropriate preclinical models of HD. Nevertheless, our PAS-based haplotype-targeting CRISPR/Cas9 strategy targeting the root cause of the disease selectively and permanently may overcome key limitations of other gene-lowering approaches and, therefore, has the potential of being tested in CRISPR/Cas9 intervention trials for HD and others.

## Methods

### Identification of exonic PAS on HTT to design allele-specific CRISPR/Cas9 strategies for HD.

Detailed methods for identifying PAS on *HTT* were described previously (10). In this study, we focused on revealing PAS on coding exons of *HTT* (RefSeq, NM_002111) whose reference or alternative alleles generate or eliminate the PAM sequence for WT *Sp*Cas9 (i.e., NGG). For this, we analyzed the KGP data (phase III), identifying 157 PAS residing on the exons of *HTT*. Subsequently, we excluded PAS on untranslated regions, revealing 91 PAS on the coding sequence (CDS) of *HTT* that may allow mutant *HTT*-specific NMD through CRISPR/Cas9 (Supplemental Table 1). Among 91 PAS, reference and alternative alleles of 71 and 23 SNPs generate NGG PAM sites, respectively. Both reference and alternative alleles of 3 PAS (rs545932099, rs1065747, rs148032171) generate NGG PAM sites on both plus and minus strands. The locations of PAS-generated NGG PAM sites are summarized in Supplemental Figure 1.

### Mapping PAS-generated NGG PAM sites on the common HTT haplotypes.

Aiming at developing mutant-specific CRISPR/Cas9 strategies that do not directly target the expanded CAG repeat but, rather, the mutant *HTT* haplotype carrying an expanded CAG repeat, we identified PAS-generated NGG PAM sites on common *HTT* haplotypes in HD using phased KGP genotype data. We determined the *HTT* haplotype of each chromosome in the phased KGP data set using our haplotype definitions (14), focusing on the 8 common haplotypes that, together, account for more than 80% of the mutant chromosomes in HD subjects with European ancestry (14, 17, 89). Subsequently, we grouped KGP chromosomes of the same *HTT* haplotypes together, revealing 103, 217, 225, 11, 16, 196, 25, and 794 KGP chromosomes for hap.01, hap.02, hap.03, hap.04, hap.05, hap.06, hap.07, and hap.08, respectively. For a given haplotype, we then identified consensus alleles by taking the most frequent alleles of PAS to generate a map of PAS-generated PAM sites on each haplotype (summarized in Supplemental Figure 2). We then performed a pairwise comparison to reveal polymorphic or haplotype-specific PAS-generated PAM sites.

### Cell culture.

Two independent iPSC lines (iPSC-A and iPSC-B) carrying adult-onset CAG repeats (46 CAG8 and 42 CAG, respectively) were derived from our internal collection of HD lymphoblastoid cell lines (LCL) by the Harvard Stem Cell Institute iPS Core Facility (http://ipscore.hsci.harvard.edu/). Both iPSC-A (female) and iPSC-B (male) carry expanded and normal CAG repeats on hap.01 and hap.08 haplotypes, respectively. iPSC-A and iPSC-B were mainly used to determine allele specificities and molecular outcomes of our mutant-specific CRISPR/Cas9 strategy. To determine the impacts of allele-specific CRISPR/Cas9 on HTT protein levels, an iPSC line with a juvenile-onset CAG repeat (iPSC-C; 72/15 CAGs; female) (90) was generated from GM04723 (https://catalog.coriell.org/0/Sections/Search/Sample_Detail.aspx?Ref=GM04723&Product=CC) (71) by the Harvard Stem Cell Institute iPS Core Facility (http://ipscore.hsci.harvard.edu/). A non-HD iPSC line from Coriell GM08330 (https://catalog.coriell.org/0/Sections/Search/Sample_Detail.aspx?Ref=GM08330&Product=CC) was analyzed as a control. NPC lines from GM09197 (https://catalog.coriell.org/0/Sections/Search/Sample_Detail.aspx?Ref=GM09197&Product=CC) and GM04723 (https://catalog.coriell.org/0/Sections/Search/Sample_Detail.aspx?Ref=GM04723&Product=CC) are described elsewhere (10, 71, 76). Each HD iPSC carries one expanded (CAG > 35) and one normal repeat (CAG < 36) (i.e., heterozygous). iPSC lines were cultured on Matrigel-coated plates (Corning) with mTeSR1 (Stemcell Technologies) with 5% CO_2_ at 37°C. NPC lines were generated from the iPSC lines by a STEMdiff protocol using Neural Induction Medium (Stemcell Technologies). These NPC cell lines were maintained on Matrigel-coated plates in media (70% DMEM, 30% Hams F12, 1× B27 Supplement, 20 ng/mL FGF, 20 ng/mL EGF, and 5 μg/mL Heparin) with 5% CO_2_ at 37°C.

### Plasmid for SpCas9, gRNA cloning, and transfection.

PX551 (http://n2t.net/addgene:60957) and PX552 (http://n2t.net/addgene:60958) were obtained from Addgene. To express *Sp*Cas9 in iPSC lines, the pMecp2 promoter in the original PX551 plasmid was replaced by an EF-1α core promoter using HindIII/AgeI fragment, generating PX551 EFS plasmid. The hSyn promoter was also replaced by an EF-1α core promoter using ApaI/KpnI fragment to express EGFP marker (PX552 EFS plasmid). Cloning of the target test gRNA into the PX552 EFS plasmid was performed according to a recommended protocol (https://media.addgene.org/data/plasmids/60/60958/60958-attachment_wWVpb-8u9Mzp.pdf). The lentiCRISPRv2 plasmid (Addgene plasmid 52961; https://www.addgene.org/52961/) was used to develop single cell clones from a juvenile onset HD iPSC (iPSC-C, 72 CAG). Sequences of oligos to generate the plasmids are summarized in Supplemental Table 8. Cells were transfected with either (a) *Sp*Cas9 plasmid and EV for gRNA (EV control) or (b) *Sp*Cas9 plasmid and gRNA plasmid (treatment group) by electroporation using Human Stem Cell Nucleofector 1 (Lonza) or Lipofectamine Stem Transfection Reagent (Invitrogen) according to manufacturer instructions.

### Nucleic acid isolation and PCR.

Genomic DNA was isolated from cells using DNeasy Blood & Tissue Kit (Qiagen). Total RNA was extracted using RNeasy Plus Mini Kits (Qiagen). The quality and quantity of RNA were determined using a NanoDrop spectrophotometer (Thermo Fisher Scientific). cDNA was synthesized from 50 ng of total RNA using SuperScript IV Reverse Transcriptase (Invitrogen). PCR reactions were performed using Q5 High-Fidelity DNA Polymerase (NEB) or AccuPrime GC-Rich DNA Polymerase (Invitrogen) for high GC content PCR. The primer sets used in PCR experiments are listed in Supplemental Table 8. The PCR amplification using Q5 High-Fidelity DNA Polymerase consisted of initial denaturation for 3 minutes at 98°C, 35 cycles of denaturation for 30 seconds at 98°C, annealing for 30 seconds at 64°C, and extension for 30 seconds at 72°C; final extension for 2 minutes at 72°C was followed by cooling down to 4°C. The reaction for AccuPrime GC-Rich DNA Polymerase consisted of an initial denaturation for 3 minutes at 95°C, followed by 30 cycles of denaturation for 30 seconds at 95°C, annealing for 30 seconds at 59°C, and extension for 30 seconds at 72°C; final extension lasted for 10 minutes at 72°C, followed by cooling down to 4°C. PCR products were purified using QIAquick PCR Purification Kit (Qiagen) for subsequent analysis.

### MiSeq analysis of PCR amplicons.

Upon ligation of Illumina adaptors and a unique identifier to the amplicon, paired-end sequencing (2 × 150 bp) was performed by the Illumina MiSeq platform. Deep sequencing of PCR amplicons was performed by the DNA Core Facility at Massachusetts General Hospital (https://dnacore.mgh.harvard.edu/new-cgi-bin/site/pages/index.jsp). Sequence reads that could not be mapped were removed as part of quality control. Primers for PCR amplification are listed in Supplemental Table 8.

### Generation of targeted single-cell clonal lines.

To generate single-cell clones from iPSC derived from GM04723 (i.e., iPSC-C), cells were transfected by electroporation with a lentiCRISPRv2 vector expressing only *Sp*Cas9 or a plasmid expressing both *Sp*Cas9 and gRNA. Seventy-two hours after puromycin selection (0.5 mg/L), 1.5 × 10^5^ cells were seeded in a 60 mm dish with 10 μM ROCK inhibitor (MilliporeSigma) and then incubated for 2 weeks without ROCK inhibitor. To generate single-cell clones from iPSCs with adult-onset CAG repeats (iPSC-A and iPSC-B), cells were transfected with PX551 EFS and PX552 EFS vectors (containing either EV or the target gRNA) using Lipofectamine Stem Transfection Reagent. Seventy-two hours after transfection without selection, 1.5 × 10^5^ cells were seeded in a 60 mm dish with CloneR supplement (Stemcell Technologies) and then incubated for an additional 2 weeks. Visible colonies were picked and individually maintained in 96-well plates for clonal expansion. Once cells reached approximately 80% confluent, cells were subcultured in two 96-well plates, one for maintenance and the other for genomic DNA extraction to validate the on-target modification by Sanger sequencing analysis. Confirmed targeted clones were then expanded for molecular characterization and RNA-Seq analysis.

### Western blot analysis.

Cells were washed twice with cold PBS and lysed with RIPA buffer (Invitrogen) containing protease inhibitor (Roche). Cell debris were removed by centrifugation (17,900*g*, 15 minutes, 4°C), and the supernatant was collected. After protein concentration was measured by BCA assays (Thermo Fisher Scientific), samples were prepared by denaturing the lysate in 2× SDS buffer (Invitrogen) with a reducing agent (Invitrogen) for 2 minutes at 80°C. In total, 15 μg of whole-cell lysate was resolved on a 6% Tris-Glycine gel (Invitrogen) unless stated otherwise. Transferred membranes were probed by a mutant huntingtin-specific antibody (1F8) (91, 92) or panhuntingtin antibody such as MAB2166 (MilliporeSigma; aa 181–810; catalog MAB2166) and N17 antibody provided in-house (93). Equal loading was confirmed by either α-tubulin antibody (Santa Cruz Biotechnology Inc., sc-5287) or β-actin antibody (MilliporeSigma, A2228).

### RNA-Seq analysis.

Detailed procedures are described in Supplemental Methods. RNA-Seq data of control and targeted iPSC clones have been deposited in Dryad (citation https://doi.org/10.5061/dryad.1g1jwstsb).

### GUIDE-Seq.

Detailed methods are described in Supplemental Methods. Sequence information is described in Supplemental Table 9.

### Off-target prediction and subsequent enrichment analysis using RNA-Seq data.

Detailed procedures are described in Supplemental Methods.

### Allele-specific CRISPR/Cas9 targeting in differentiated neurons from patient-derived iPSC.

Detailed procedures are described in Supplemental Methods.

### Genomic coordinate.

Genomic coordinate is based on GRCh37/hg19.

### Statistics.

Statistical significances were determined by Student *t* tests (2 tailed) and linear regression analyses. Resulting nominal *P* values were corrected for multiple tests by Bonferroni and FDR method for ASE and DGE analysis, respectively. Corrected *P* values less than 0.05 were considered statistically significant.

### Study approval.

Patient consents and the overall study for the genome-wide association study were previously described (18). Patient-derived cells were obtained from a public repository.

## Author contributions

JML and JWS designed the study. JWS, SSP, and DEC performed experiments. EPH performed bioinformatic analysis. MNW and BPK performed GUIDE-Seq experiments and analyzed data. JWS, EPH, ISS, MNW, BPK, RZC, and JML wrote the manuscript.

## Supplementary Material

Supplemental data

## Figures and Tables

**Table 1:**
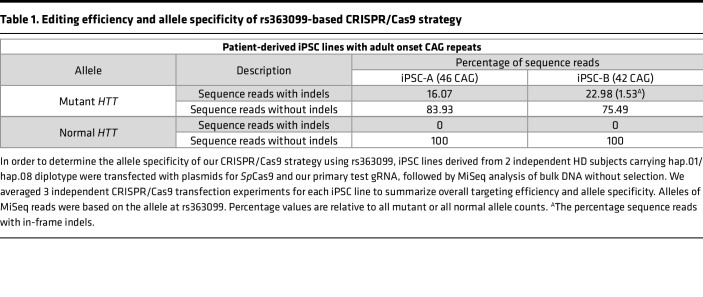
Editing efficiency and allele specificity of rs363099-based CRISPR/Cas9 strategy

## Version 1 08/30/2022

In-Press Preview

## Version 2 10/10/2022

Electronic publication

## References

[1] [No authors listed] (1993). A novel gene containing a trinucleotide repeat that is expanded and unstable on Huntington’s disease chromosomes. The Huntington’s disease collaborative research group. Cell.

[2] Bates GP (2015). Huntington disease. Nat Rev Dis Primers.

[3] Lee JM (2012). CAG repeat expansion in Huntington disease determines age at onset in a fully dominant fashion. Neurology.

[4] Genetic Modifiers of Huntington’s Disease (GeM-HD) Consortium (2015). Identification of genetic factors that modify clinical onset of Huntington’s disease. Cell.

[6] Walker FO (2007). Huntington’s disease. Lancet.

[7] Nasir J (1995). Targeted disruption of the Huntington’s disease gene results in embryonic lethality and behavioral and morphological changes in heterozygotes. Cell.

[8] White JK (1997). Huntingtin is required for neurogenesis and is not impaired by the Huntington’s disease CAG expansion. Nat Genet.

[9] Zeitlin S (1995). Increased apoptosis and early embryonic lethality in mice nullizygous for the Huntington’s disease gene homologue. Nat Genet.

[10] Shin JW (2016). Permanent inactivation of Huntington’s disease mutation by personalized allele-specific CRISPR/Cas9. Hum Mol Genet.

[11] Smits AH (2019). Biological plasticity rescues target activity in CRISPR knock outs. Nat Methods.

[12] Kurosaki T (2019). Quality and quantity control of gene expression by nonsense-mediated mRNA decay. Nat Rev Mol Cell Biol.

[13] Baine FK (2013). Huntington disease in the South African population occurs on diverse and ethnically distinct genetic haplotypes. Eur J Hum Genet.

[14] Chao MJ (2017). Haplotype-based stratification of Huntington’s disease. Eur J Hum Genet.

[15] Warby SC (2011). HTT haplotypes contribute to differences in Huntington disease prevalence between Europe and East Asia. Eur J Hum Genet.

[16] Shin JW (2022). Haplotype-specific insertion-deletion variations for allele-specific targeting in Huntington’s disease. Mol Ther Methods Clin Dev.

[17] Lee JM (2012). Common SNP-based haplotype analysis of the 4p16.3 Huntington disease gene region. Am J Hum Genet.

[18] Genetic Modifiers of Huntington’s Disease (GeM-HD) Consortium (2019). CAG repeat not polyglutamine length determines timing of Huntington’s disease onset. Cell.

[19] Monteys AM (2017). CRISPR/Cas9 editing of the mutant huntingtin allele in vitro and in vivo. Mol Ther.

[20] Yang S (2017). CRISPR/Cas9-mediated gene editing ameliorates neurotoxicity in mouse model of Huntington’s disease. J Clin Invest.

[21] Chakrabarti AM (2019). Target-specific precision of CRISPR-mediated genome editing. Mol Cell.

[22] Deng HX (1993). Amyotrophic lateral sclerosis and structural defects in Cu,Zn superoxide dismutase. Science.

[23] Goate A (1991). Segregation of a missense mutation in the amyloid precursor protein gene with familial Alzheimer’s disease. Nature.

[24] Rosen DR (1993). Mutations in Cu/Zn superoxide dismutase gene are associated with familial amyotrophic lateral sclerosis. Nature.

[25] Tanzi RE (1992). Assessment of amyloid beta-protein precursor gene mutations in a large set of familial and sporadic Alzheimer disease cases. Am J Hum Genet.

[26] Heidenreich M, Zhang F (2016). Applications of CRISPR-Cas systems in neuroscience. Nat Rev Neurosci.

[27] Jackson AL (2006). Widespread siRNA “off-target” transcript silencing mediated by seed region sequence complementarity. RNA.

[28] Scacheri PC (2004). Short interfering RNAs can induce unexpected and divergent changes in the levels of untargeted proteins in mammalian cells. Proc Natl Acad Sci U S A.

[29] Persengiev SP (2004). Nonspecific, concentration-dependent stimulation and repression of mammalian gene expression by small interfering RNAs (siRNAs). RNA.

[30] Jackson AL (2003). Expression profiling reveals off-target gene regulation by RNAi. Nat Biotechnol.

[31] Tabrizi SJ (2019). Huntingtin lowering strategies for disease modification in Huntington’s disease. Neuron.

[32] Mercuri E (2018). Nusinersen versus sham control in later-onset spinal muscular atrophy. N Engl J Med.

[33] Finkel RS (2017). Nusinersen versus sham control in infantile-onset spinal muscular atrophy. N Engl J Med.

[34] Bonini NM, La Spada AR (2005). Silencing polyglutamine degeneration with RNAi. Neuron.

[35] Boudreau RL (2009). Nonallele-specific silencing of mutant and wild-type huntingtin demonstrates therapeutic efficacy in Huntington’s disease mice. Mol Ther.

[36] Carroll JB (2011). Potent and selective antisense oligonucleotides targeting single-nucleotide polymorphisms in the Huntington disease gene / allele-specific silencing of mutant huntingtin. Mol Ther.

[37] Keiser MS (2015). Gene suppression strategies for dominantly inherited neurodegenerative diseases: lessons from Huntington’s disease and spinocerebellar ataxia. Hum Mol Genet.

[38] Smith RA (2006). Antisense oligonucleotide therapy for neurodegenerative disease. J Clin Invest.

[39] Yu D (2012). Single-stranded RNAs use RNAi to potently and allele-selectively inhibit mutant huntingtin expression. Cell.

[40] Tabrizi SJ (2019). Targeting huntingtin expression in patients with Huntington’s disease. N Engl J Med.

[41] Sheridan C (2021). Questions swirl around failures of disease-modifying Huntington’s drugs. Nat Biotechnol.

[42] Keiser MS (2016). Gene suppression strategies for dominantly inherited neurodegenerative diseases: lessons from Huntington’s disease and spinocerebellar ataxia. Hum Mol Genet.

[43] Johnson CD, Davidson BL (2010). Huntington’s disease: progress toward effective disease-modifying treatments and a cure. Hum Mol Genet.

[44] Wild EJ, Tabrizi SJ (2017). Therapies targeting DNA and RNA in Huntington’s disease. Lancet Neurol.

[45] Stadtmauer EA (2020). CRISPR-engineered T cells in patients with refractory cancer. Science.

[46] Jain A (2017). CRISPR-Cas9-based treatment of myocilin-associated glaucoma. Proc Natl Acad Sci U S A.

[47] Long C (2014). Prevention of muscular dystrophy in mice by CRISPR/Cas9-mediated editing of germline DNA. Science.

[48] Dietrich P (2017). Elimination of huntingtin in the adult mouse leads to progressive behavioral deficits, bilateral thalamic calcification, and altered brain iron homeostasis. PLoS Genet.

[49] Dragatsis I (2000). Inactivation of Hdh in the brain and testis results in progressive neurodegeneration and sterility in mice. Nat Genet.

[50] Wang G (2016). Ablation of huntingtin in adult neurons is nondeleterious but its depletion in young mice causes acute pancreatitis. Proc Natl Acad Sci U S A.

[51] Lopes F (2016). Identification of novel genetic causes of Rett syndrome-like phenotypes. J Med Genet.

[52] Rodan LH (2016). A novel neurodevelopmental disorder associated with compound heterozygous variants in the huntingtin gene. Eur J Hum Genet.

[53] Ambrose CM (1994). Structure and expression of the Huntington’s disease gene: evidence against simple inactivation due to an expanded CAG repeat. Somat Cell Mol Genet.

[54] Huntington G (1872). On chorea. Med Surg Rep.

[55] Paulsen JS (2008). Detection of Huntington’s disease decades before diagnosis: the predict-HD study. J Neurol Neurosurg Psychiatry.

[56] Walton RT (2020). Unconstrained genome targeting with near-PAMless engineered CRISPR-Cas9 variants. Science.

[57] Kleinstiver BP (2019). Engineered CRISPR-Cas12a variants with increased activities and improved targeting ranges for gene, epigenetic and base editing. Nat Biotechnol.

[58] Ma D (2019). Engineer chimeric Cas9 to expand PAM recognition based on evolutionary information. Nat Commun.

[59] Nishimasu H (2018). Engineered CRISPR-Cas9 nuclease with expanded targeting space. Science.

[60] Hirano S (2016). Structural basis for the altered PAM specificities of engineered CRISPR-Cas9. Mol Cell.

[61] Kleinstiver BP (2015). Broadening the targeting range of Staphylococcus aureus CRISPR-Cas9 by modifying PAM recognition. Nat Biotechnol.

[62] Kleinstiver BP (2015). Engineered CRISPR-Cas9 nucleases with altered PAM specificities. Nature.

[63] Duong TT (2019). Comparative AAV-eGFP transgene expression using vector serotypes 1-9, 7m8, and 8b in human pluripotent stem cells, RPEs, and human and rat cortical neurons. Stem Cells Int.

[64] Tian R (2019). CRISPR interference-based platform for multimodal genetic screens in human iPSC-derived neurons. Neuron.

[65] Iwamoto M (2010). A general chemical method to regulate protein stability in the mammalian central nervous system. Chem Biol.

[66] Zuckermann M (2015). Somatic CRISPR/Cas9-mediated tumour suppressor disruption enables versatile brain tumour modelling. Nat Commun.

[67] Swiech L, et al F (2015). In vivo interrogation of gene function in the mammalian brain using CRISPR-Cas9. Nat Biotechnol.

[68] Incontro S (2014). Efficient, complete deletion of synaptic proteins using CRISPR. Neuron.

[69] Straub C, et al 2014 (2014). CRISPR/Cas9-mediated gene knock-down in post-mitotic neurons. PLoS One.

[70] Koyuncu S (2018). The ubiquitin ligase UBR5 suppresses proteostasis collapse in pluripotent stem cells from Huntington’s disease patients. Nat Commun.

[71] HD iPSC Consortium (2012). Induced pluripotent stem cells from patients with Huntington’s disease show CAG-repeat-expansion-associated phenotypes. Cell Stem Cell.

[72] Victor MB (2018). Striatal neurons directly converted from Huntington’s disease patient fibroblasts recapitulate age-associated disease phenotypes. Nat Neurosci.

[73] Conforti P (2018). Faulty neuronal determination and cell polarization are reverted by modulating HD early phenotypes. Proc Natl Acad Sci U S A.

[74] Chae JI (2012). Quantitative proteomic analysis of induced pluripotent stem cells derived from a human Huntington’s disease patient. Biochem J.

[75] HD iPSC Consortium (2017). Developmental alterations in Huntington’s disease neural cells and pharmacological rescue in cells and mice. Nat Neurosci.

[76] Mattis VB (2015). HD iPSC-derived neural progenitors accumulate in culture and are susceptible to BDNF withdrawal due to glutamate toxicity. Hum Mol Genet.

[77] Le Cann K (2021). The difficulty to model Huntington’s disease in vitro using striatal medium spiny neurons differentiated from human induced pluripotent stem cells. Sci Rep.

[78] Jeon I (2012). Neuronal properties, in vivo effects, and pathology of a Huntington’s disease patient-derived induced pluripotent stem cells. Stem Cells.

[79] Seriola A (2011). Huntington’s and myotonic dystrophy hESCs: down-regulated trinucleotide repeat instability and mismatch repair machinery expression upon differentiation. Hum Mol Genet.

[80] Niclis J (2009). Human embryonic stem cell models of Huntington disease. Reprod Biomed Online.

[81] Southwell AL (2013). A fully humanized transgenic mouse model of Huntington disease. Hum Mol Genet.

[82] Cullot G (2019). CRISPR-Cas9 genome editing induces megabase-scale chromosomal truncations. Nat Commun.

[83] Zhang L (2015). Large genomic fragment deletions and insertions in mouse using CRISPR/Cas9. PLoS One.

[84] Scott DA, Zhang F (2017). Implications of human genetic variation in CRISPR-based therapeutic genome editing. Nat Med.

[85] Li XL (2018). Highly efficient genome editing via CRISPR-Cas9 in human pluripotent stem cells is achieved by transient BCL-XL overexpression. Nucleic Acids Res.

[86] Yang L (2013). Optimization of scarless human stem cell genome editing. Nucleic Acids Res.

[87] Mali P (2013). CAS9 transcriptional activators for target specificity screening and paired nickases for cooperative genome engineering. Nat Biotechnol.

[88] Liu H (2021). Huntingtin silencing delays onset and slows progression of Huntington’s disease: a biomarker study. Brain.

[89] Lee JM (2015). Sequence-level analysis of the major European Huntington disease haplotype. Am J Hum Genet.

[90] Kim KH (2020). Genetic and functional analyses point to FAN1 as the source of multiple Huntington disease modifier effects. Am J Hum Genet.

[91] Persichetti F (1999). Mutant huntingtin forms in vivo complexes with distinct context-dependent conformations of the polyglutamine segment. Neurobiol Dis.

[92] Huang CC (1998). Amyloid formation by mutant huntingtin: threshold, progressivity and recruitment of normal polyglutamine proteins. Somat Cell Mol Genet.

[93] Sapp E (2012). Native mutant huntingtin in human brain: evidence for prevalence of full-length monomer. J Biol Chem.

