# Supplementary material for: Allele-specific silencing of the gain-of-function mutation in Huntington’s disease using CRISPR/Cas9
Source: JCI Insight. 2022 Oct 10;7(19):e141042. doi: 10.1172/jci.insight.141042 (PMC9675467; doi:10.1172/jci.insight.141042)
Supplement: Supplemental data [file jciinsight-7-141042-s126.pdf]

Supplemental Methods

RNAseq analysis. To fully characterize the molecular consequences of our mutant-specific CRISPR-Cas9 strategy, we performed RNAseq analysis. We transfected two HD iPSC lines (iPSC-A and iPSC-B) carrying adult onset CAG repeats with mutant-specific CRISPR-Cas9 (PX551 EFS vector expression *Sp*Cas9 and PX552 EFS plasmid expressing our test gRNA; experimental group) or empty vector (PX551 EFS vector for *Sp*Cas9 and empty PX552 vector without gRNA; control group). Single cell clones were subsequently developed by limited dilution. 12 clonal lines were developed for each group and further validated by Sanger sequencing and MiSeq analysis of genomic DNA (PCR primers for Sanger sequencing and MiSeq analysis are listed in Supplemental Table 8). Then, genome-wide RNAseq analysis was performed by the Broad Institute. Sequence data were processed by STAR aligner (1) as part of the Broad Institute's standard RNAseq analysis pipeline.

For allele-specific expression analysis (ASE) of RNAseq data focusing on *HTT*¸ we counted alleles harboring heterozygous exonic SNPs in targeted and control clonal lines. Our clonal lines derived from two HD iPSC with hap.01/hap.08 diplotype carry 10 heterozygous exonic SNPs including rs363099. Alleles of 10 heterozygous exonic SNPs on the mutant and normal *HTT* in hap.01/hap.08 diplotype were determined based on haplotype sequence data (2). Statistical significance in ASE of *HTT* was judged based on the Student t-test (2-tailed), comparing counts of alleles on normal *HTT* in targeted clonal lines (n=12) to those in controls (n=12) for a given SNP site, followed by Bonferroni multiple test correction (Figure 4B). The same approach was applied to the numbers of alleles on the mutant *HTT* to compare targeted and control clones (Figure 4C).

For differential gene expression (DGE) analysis, we used transcripts per million (TPM) data computed by the TPMCalculator (https://github.com/ncbi/TPMCalculator) (3). Expression levels in 20,260 protein-coding genes based on Ensembl (ftp://ftp.ensembl.org/pub/release-75/gtf/homo_sapiens/) were normalized; 3,420 genes were excluded due to zero TPM values in at least one sample. Subsequently, we analyzed 16,840 genes expressed in all 24 samples. The DGE analysis was performed by the generalized linear model using a library of “glm” in R package v3.3.1 (https://www.r-project.org/) after adjustment for four covariates including sex, batch, and two principal components based on RNAseq data, followed by multiple test correction using a FDR method. A multiple test corrected p-value less than 0.05 was considered statistically significant.

Off-target prediction and subsequent enrichment analysis using RNAseq data. Potential off-targets of our primary test gRNA were based on the Cas-OFFinder website (http://www.rgenome.net/cas-offinder/). Parameters for Off-target prediction are maximum 2 mismatches, 1 bulge in DNA, or 1 bulge in RNA. A total of 77 unique potential off-targets were predicted, including 1 exonic and 30 intronic sites of protein coding genes. When a predicted off-target is located between genes, we took the nearest gene to represent the predicted off-target in DGE analysis and gene set enrichment analysis of RNAseq data. As a result, 77 predicted off-target sites were mapped to 52 expressed genes in our RNAseq data. For gene set enrichment analysis of predicted off-targets, we calculated true gene set score by summing the significance values (-log10(FDR)) of predicted

off-targets, and compared to a null distribution of enrichment score based on 1 million random samplings to obtain an empirical p-value.

GUIDE-seq. HEK 293T cells (ATCC CRL-3216) were cultured in Dulbecco's Modified Eagle Medium (DMEM) supplemented with 10% heat-inactivated FBS (HI-FBS) and 1% penicillin/streptomycin. Transfections of HEK 293T cells were performed between 18 and 24 hours following seeding of approximately 20,000 cells per well in 96-well plates. Transfections contained 29 ng of nuclease and 12.5 ng of single guide RNA (sgRNA) expression plasmids, 1 pmol of the GUIDE-seq double-stranded oligodeoxynucleotide (dsODN; oSQT685/686) tag (4), and 0.3 µL of TransIT-X2 (Muris). The transfection components were combined in a total volume of 16 µL supplemented with Opti-MEM, incubated at room temperature for 15 minutes, and then gently added to the media of the plated cells. Genomic DNA from the transfected cells was harvested approximately 72 hours post-transfection by discarding the media and re-suspending the cells in 100 µL of lysis buffer (20 mM Hepes pH 7.5, 100 mM KCl, 5 mM MgCl2, 5% glycerol, 25 mM DTT, 0.1% Triton X-100, and 60 ng/µL Proteinase K). The lysate was heated for 6 minutes at 65 ºC, 98 ºC for 2 minutes, and then stored at -20 ºC until use. To perform the GUIDE-seq assay, the efficiency of on-target editing and GUIDE-seq dsODN tag capture was first assessed by sequencing via a 2-step PCR-based Illumina library preparation method. Genomic loci were amplified by a first PCR using Q5 High-fidelity DNA Polymerase (NEB) and the primers listed in Supplemental Table 9. Products from PCR #1 were quantified via capillary electrophoresis (Qiagen QIAxcel) and purified via paramagnetic beads (5, 6). A second PCR reaction was performed to add Illumina barcodes and adapter sequences using approximately 20 pg of purified PCR #1 as the template. Final sequencing libraries were quantified by qPCR (Illumina Library qPCR Quantification Kit, KAPA Biosystems), then sequenced on a MiSeq sequencer using a 300 cycle Micro Kit v2 (Illumina). On-target tag capture was quantified using CRISPResso2 (https://crispresso.pinellolab.partners.org/submission) (7) by including sequences for the target site spacer, the reference amplicon, the expected forward and reverse orientation dsODN containing amplicons as 'HDR' alleles, and the following custom parameters: -w 25 -g GUIDE --plot_window_size 50. Total dsODN capture was quantified by dividing the sum of the forward and reverse dsODN-containing reads (modified and unmodified) reads by the total number of reads for the sample. To analyze potential off-target editing events, GUIDE-seq sequencing libraries were prepared from the samples of genomic DNA (5, 6). Briefly, genomic DNA was purified from the cell lysates using a 1X ratio of paramagnetic beads, quantified via QuantiFluor (Promega), and 400ng of genomic DNA from each sample was normalized to a final volume of 130 µL using 1x TE buffer. Samples were sheared to 500 bp using an ultrasonicator (Covaris E220) and sequencing libraries were prepared as previously described (5, 6) using the sample barcodes listed in Supplemental Table 9. Samples were sequenced on an Illumina MiSeq using a 300 cycle v2 kit with custom Read 2 and Index 1 sequencing primers (Supplemental Table 9). The resulting GUIDE-seq data were analyzed using guideseq v1.0.2 (https://github.com/aryeelab/guideseq) with a mismatch tolerance of 6 (max_mismatches 6).

Allele-specific CRISPR-Cas9 targeting in differentiated neurons from patient-derived iPSC. An iPSC line with an adult onset CAG repeat (iPSC-B, 42 CAG) was differentiated into neuronal cells using a previously

described method (8). An iPSC-B line was plated on growth factor reduced Matrigel (Corning) in mTeSR Plus media (STEMCELL Technologies). When cells reached ~ 80% confluence, differentiation was initiated by switching to DMEM-F12/Neurobasal media (2:1) supplemented with N2 and retinol-free B27 (N2B27 RA−; Gibco). For the first ten days, cells were supplemented with SB431542 (10 μM; Tocris), LDN-193189 (100 nM; StemGene), and dorsomorphin (200 nM; Tocris). SB431542 was removed from the media on day 5. Cells were maintained in N2B27 RA− supplemented with activin A (25 ng/ml; R&D) on day 9. On day 22, cells were split using Accutase (STEMCELL Technologies) and seeded on a poly-D-lysine/laminin plate with N2B27 media supplemented with BDNF and GDNF (10 ng/ml each; Peprotech). Media were changed the next day to facilitate neuronal maturation and survival. Cells were fed with new media every two days.

In order to check the expression levels of marker genes, total RNA of iPSC-B and iPSC-B-derived neuronal cells were extracted using RNeasy Plus Kits (Qiagen), and reverse transcription was performed with SuperScript IV First-Strand Synthesis System (Invitrogen). One μl cDNA samples and following primers were used for the PCR amplification for OCT4 (octamer binding transcription factor 4; 5'-GACACCCCCACCCCACCAGCTAGG-3' and 5'-CTTCCCTCCAACCAGTTGCCCCAAA-3), MAP2 (microtubule-associated protein 2; 5'-CTGCTTTACAGGGTAGCACAA-3' and 5'-TTGAGTATGGCAAACGGTCTG-3'), GAD67 (glutamic acid decarboxylase 67; 5'-CGTCTTCGACCCCATCTTCGT-3' and 5'-CGCAGATCTTGAGCCCCAGTT-3'), TH (tyrosine hydroxylase; 5'-GAGTACACCGCCGCCGAGGAGATTG-3' and 5'-GCGGATATACTGGGTGCACTGG-3'), vGLUT1 (vesicular glutamate transporter 1; 5'-TACACGGCTCCTTTTTCTGG-3' and 5'-CTGAGGGGATCAGCATGTTT-3'), and GAPDH (glyceraldehyde-3-phosphate dehydrogenase; 5'-ATGACATCAAGAAGGTGGTG-3' and 5'-CATACCAGGAAATGAGCTTG-3'). Real-time PCR was performed using the SYBR Green PCR Master Mix (Applied Biosystems; denaturation at 95ºC for 5 min followed by 45 cycles of 95ºC for 10 sec, 60ºC for 10 sec, and 72ºC for 10 sec) by LightCycler 480 System (Roche). The relative expression levels were then determined using the $2^{-\Delta\Delta Ct}$ method, and fold-changes were calculated subsequently.

For neuronal marker staining, cells were fixed, permeabilized, and blocked using the Image-iT Fix-Perm kit (Invitrogen). Subsequently, cells were stainied by Anti-TUBB3 (tubline beta 3; Biolegend Inc, Cat# 801202) in a blocking solution overnight at 4℃. Then, cells were washed with PBS three times for 5 minutes, followed by incubation with Alexa Flour 594 secondary antibodies (Invitrogen) for 1 hour. Finally, cells were washed with PBS three times for 5 minutes and mounted with Vectorshield mounting medium with DAPI (Vector Laboratories). Images were captured by the Leica fluorescence microscope.

For RNP complex and mRNA transfection in differentiated neurons, Cas9 protein and Cas9 mRNA were purchased from IDT and Invitrogen, respectively. Synthesized gRNA was purchased from Invitrogen. On day 35 of neuronal differentiation, cells were transfected with either RNP complex or mRNA using Lipofectamine CRISPRMAX and Lipofectamine MessengerMAX reagent, respectively, according to the manufacturer’s instructions. After three days, genomic DNA was extracted without puromycin selection, and deep-sequencing of PCR amplicons was performed by MiSeq.

Supplemental Figure 1. Locations of exonic PAS-generated NGG PAM sites on *HTT* coding regions.

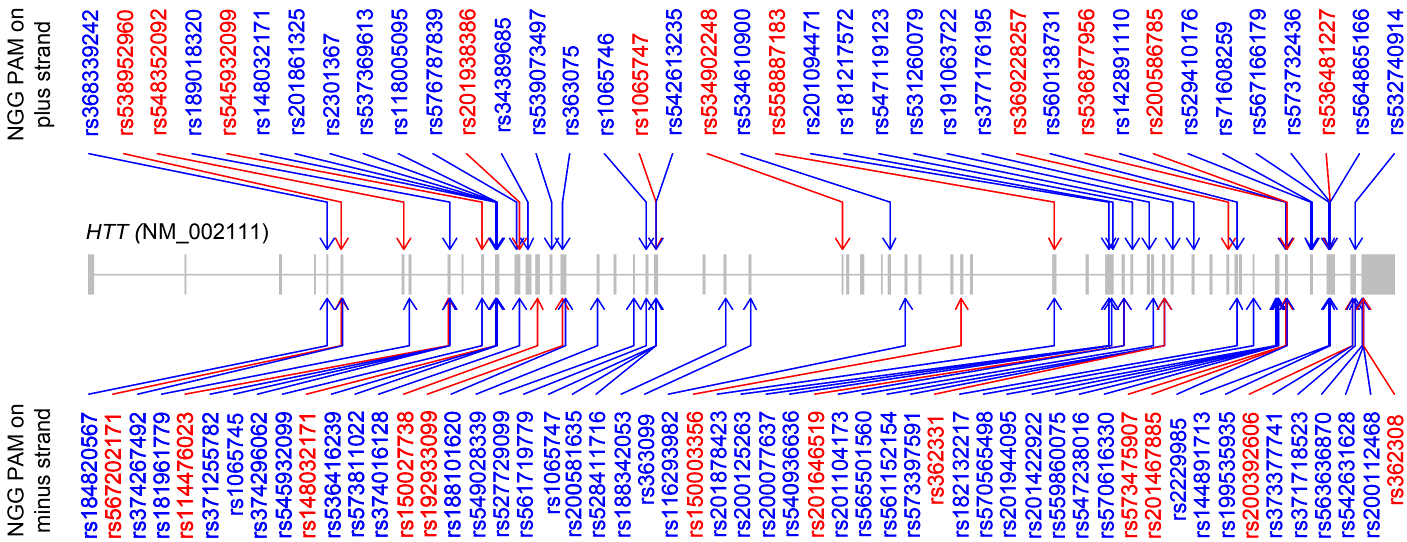

Locations of 91 PAS that are located on the coding exons of *HTT* are shown relative to RefSeq NM_002111 (grey vertical lines, exons). PAS in the top and bottom panels represent SNPs that generate NGG PAM sites on plus (39 PAS) and minus strand (55 PAS), respectively. 3 PAS (rs545932099, rs1065747, rs148032171) generate NGG PAM sites on both strands. Letters and arrows in blue and red represent NGG PAM sites generated by reference (71 PAS) and alternative alleles (23 PAS), respectively.

Supplemental Figure 2. PAS-generated NGG PAM sites on common *HTT* haplotypes.

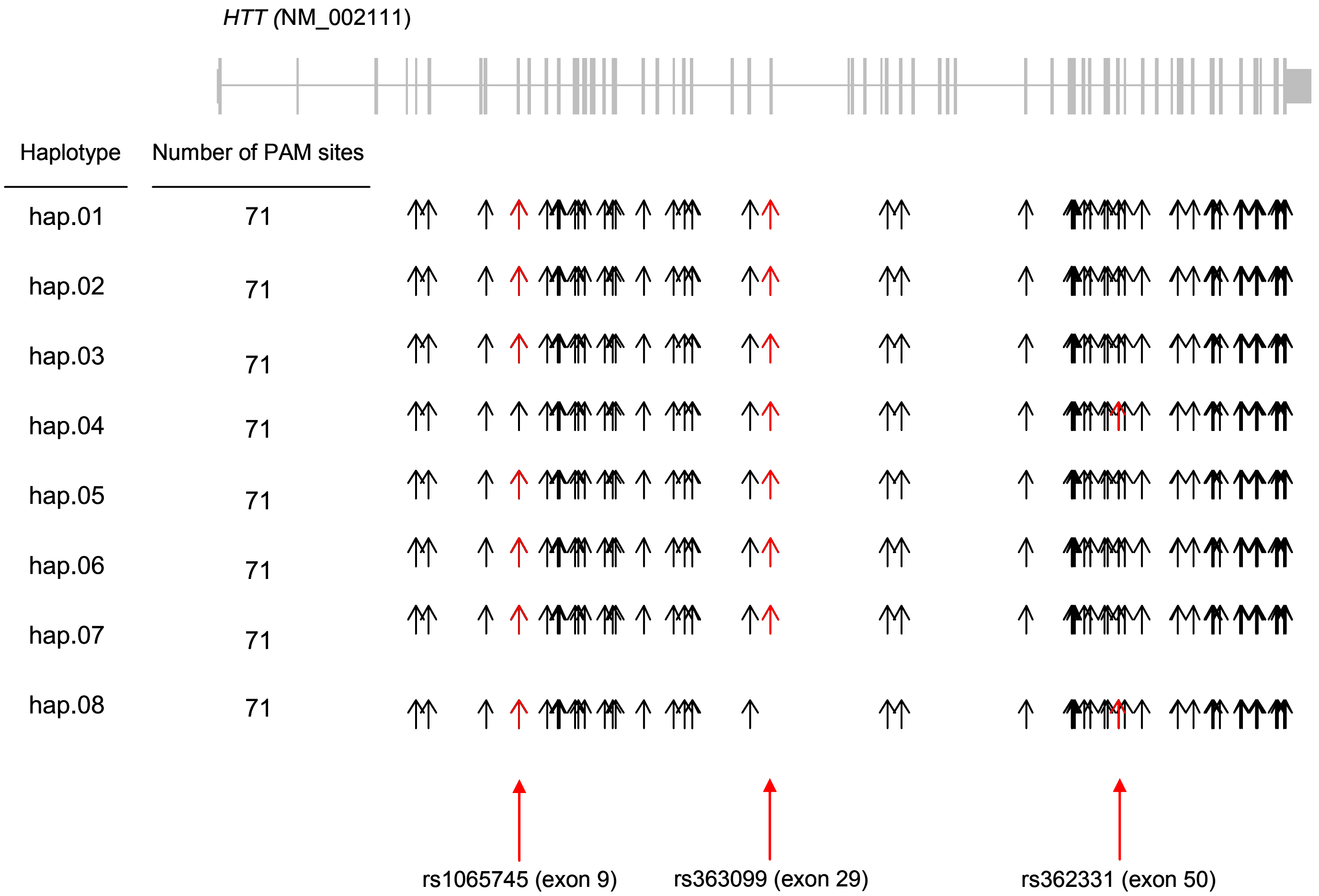

Among 91 PAS that are located on coding exons of *HTT*, 72 SNPs generate NGG PAM sites in at least one of the 8 common *HTT* haplotypes. By contrast, 19 PAS do not generate NGG PAM sites in any of 8 common *HTT* haplotypes because PAM-generating alleles of those SNPs are rare (below 1% in KGP all populations). As a result, each common haplotype carries 71 PAM sites. Among 72 SNPs that generate NGG PAM sites on common *HTT* haplotypes, 69 PAS generate NGG PAM sequence on all of 8 common *HTT* haplotypes (black arrows), and therefore they do not permit allele-specific CRISPR-Cas9 in HD subjects carrying common diplotypes. However, 3 PAS generate NGG PAM sites on some haplotypes (red arrows). For example, alternative alleles of rs1065745 (exon 9) and rs363099 (exon 29) eliminate NGG PAM site selectively on hap.04 and hap.08, respectively. In contrast, alternative allele of rs362331 (exon 50) generates NGG PAM sites selectively on hap.04 and hap.08.

Supplemental Figure 3. Targeting efficiency and allele specificity of mutant *HTT*-specific CRISPR-Cas9 in HD neural precursor cells (NPC) derived from HD patients.

A

Cell	Allele	Description	Number of reads (%)
NPCs from GM09197 (180 CAG)	Mutant	Sequence with indel	8,559 (28.2)
		Sequence w/o indel	22,209 (71.8)
	Normal	Sequence with indel	0 (0)
		Sequence w/o indel	41,829 (100)
NPCs from GM04723 (69 CAG)	Mutant	Sequence with indel	7,807 (29.2)
		Sequence w/o indel	18,963 (70.8)
	Normal	Sequence with indel	0 (0)
		Sequence w/o indel	33,192 (100)

B

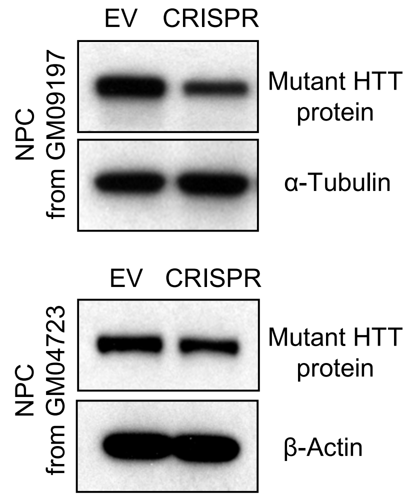

A) NPC lines derived from two juvenile onset HD patients (GM09197 and GM04723) were transfected with lentiCRISPRv2 vector expressing *Sp*Cas9 and our primary test gRNA for 72 hours without puromycin selection. Subsequently, targeting efficiency and allele frequency were determined by MiSeq analysis of bulk genomic DNA. Since both lines carry normal *HTT* on the hap.08 haplotype, each MiSeq sequencing read was determined based on the sequence at rs360399. Percentage values in parentheses are relative to either mutant or normal allele (n=1 for each NPC line). For example, 28.2% in NPC from GM09197 means 28.2% of mutant alleles contained indel modifications.

B) Whole cell lysate from NPC cell lines transfected with lentiCRISPRv2 vector expressing *Sp*Cas9 and our test gRNA for 72 hours without puromycin selection was analyzed by immunoblot analysis using 1F8 antibody to quantify the levels of mutant HTT protein. Our CRISPR-Cas9 without puromycin selection decreased 66.3% and 32.1% of mutant HTT protein levels in NPC from GM09197 (top) and GM04723 (bottom), respectively (n=1 for each NPC line). EV and CRISPR represent empty vector and CRISPR-Cas9 treatment involving our primary test gRNA, respectively.

Supplemental Figure 4. The levels of allele specificity of alternative CRISPR-Cas9 approach targeting rs363099 as gRNA hybridization.

A

rs363099

Mutant allele TTGG AGGGTTTCTCCGCTCAGCCT TGG ATGTTCTTTCTCA

gRNA hybridization PAM

Normal allele TTGG AGGGTTTCTTCGCTCAGCCT TGG ATGTTCTTTCTCA

gRNA hybridization PAM

B

iPSC-B (42 CAG)

Allele	Modification	# of reads (%)
Mutant	Sequence with indel	3,888 (18.0)
Mutant	Sequence w/o indel	17,680 (82.0)

Normal	Sequence with indel	224 (0.9)
Normal	Sequence w/o indel	26,051 (99.1)

A) In order to evaluate the levels of allele specificity of an alternative CRISPR-Cas9 approach targeting a SNP as the gRNA hybridization (not using as part of PAM site), we evaluated a different gRNA designed to hybridize to a region harboring rs363099. This gRNA utilizes a PAM site that is present on both mutant and normal *HTT* (red underlines), and therefore the PAM site for this alternative approach does not provide allele specificity. Rather, hybridization target sites in the mutant and normal alleles have zero and one mismatch with the gRNA, respectively. A red arrow and alleles in red represent rs363099. The common PAM site are underlined.

B) A patient-derived iPSC line (iPSC-B) was transfected with Cas9 and an alternative gRNA for Miseq analysis of bulk DNA. Identification of alleles was based on rs363099. Approximately, 18% of mutant *HTT* and 0.9% of normal *HTT* were edited by gRNA designed to hybridize to a region containing rs363099 (n=1).

Supplemental Figure 5. MiSeq analysis of cDNA from single cell clones targeted by our mutant-specific CRISPR-Cas9 strategy.

iPSC-C : RNA MiSeq

Targeted clonal line iPSC-C1 (23 nucleotide deletion)

rs363099

		Read count	Proportion
Normal	TTTGGAGGGT TTCTTCGCTC AGCCTTGGAT GTTCTTTCTC AGATACTAGA	16,530	93.0 %
Mutant	TTTGGAGG-- ---------- ---------- -TTCTTTCTC AGATACTAGA	1,237	7.0 %

Targeted clonal line iPSC-C2 (5 nucleotide deletion)

Normal	TTTGGAGGGT TTCTTCGCTC AGCCTTGGAT GTTCTTTCTC AGATACTAGA	55,309	93.7 %
Mutant	TTTGGAGGGT TTCTCCGC-- ---CTTGGAT GTTCTTTCTC AGATACTAGA	3,697	6.3 %

Targeted clonal line iPSC-C3 (25 nucleotide deletion)

Normal	TTTGGAGGGT TTCTTCGCTC AGCCTTGGAT GTTCTTTCTC AGATACTAGA	38,150	94.4 %
Mutant	TTTGGAGGGT TTCTC----- ---------- ---------- AGATACTAGA	2,251	5.6 %

RT-PCR assays designed to detect the CAG repeat in *HTT* mRNA revealed low levels of mutant *HTT* mRNA in the targeted clonal lines carrying a juvenile onset CAG repeat (Figure 3B). To confirm this phenomenon, we performed MiSeq analysis of cDNA from targeted single cell clones (n=3; iPSC-C1, iPSC-C2, iPSC-C3) . Total RNA was isolated from clonal lines developed by our CRISPR-Cas9 strategy utilizing a PAM site generated by rs363099, and cDNA was analyzed by PCR amplification / MiSeq analysis subsequently. Dashed lines represent deletions. T and C allele at rs363099 respectively represent alleles on the normal and mutant *HTT*. Numbers of sequence reads (Read count) and the proportion of each allele (Proportion) are shown on the right. In this analysis, a proportion value was calculated by dividing the number of mutant allele read count by the sum of mutant and normal alleles, representing the percentage of targeted mutant allele out of all sequence reads. Note, neither untargeted mutant alleles nor targeted normal alleles were detected in the genomic DNA of those clonal lines.

Supplemental Figure 6. Impacts of PAS-based mutant-specific NMD-CRISPR-Cas9 on the levels of *HTT* mRNA and protein.

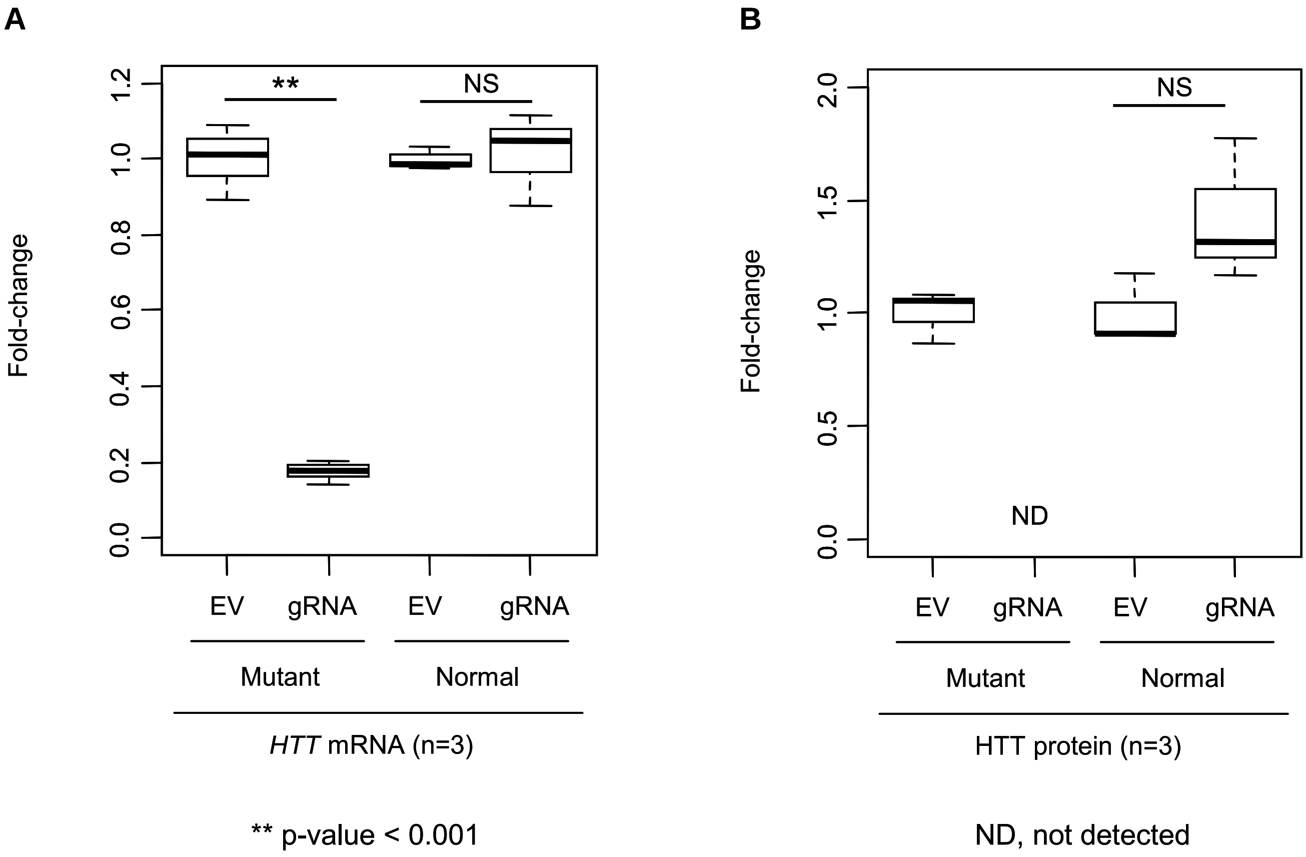

Three independent targeted clonal lines from an iPSC with a juvenile onset CAG repeat (iPSC-C, 72 CAG) were analyzed to determine the impacts of mutant *HTT*-specific NMD-CRISPR strategy on the levels of *HTT* mRNA (A) and protein (B). Band intensities of gel analysis for *HTT* mRNA (Figure 3D) and protein (Figure 3E) are shown (n=3). Mean values of the mutant and normal *HTT* in empty vector-treated clones (n=3) were set to 1 as the baseline to compare the band intensities of targeted clonal lines (n=3). Each boxplot shows the maximum, upper quarter, median, lower quarter, and minimum based on 3 independent clones/group. **, p-value < 0.001 by Student t-test (2-tailed). A p-value less than 0.05 was considered nominally significant. NS, not significant (nominal p-value > 0.05). ND, not detected.

Supplemental Figure 7. Ablation of mutant HTT protein in clonal lines targeted by our CRISPR-Cas9.

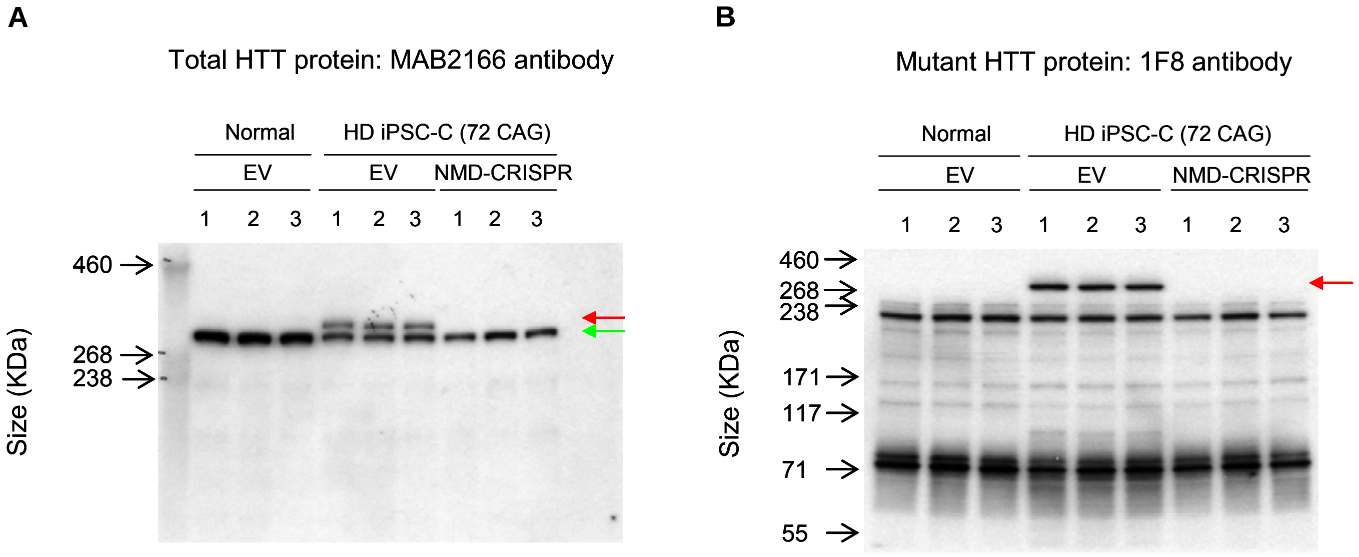

In order to determine whether our CRISPR-Cas9 selectively decreased the levels of mutant HTT protein, we performed SDS-PAGE analysis for non-HD control, EV-treated HD, and targeted clonal lines (refer to Figure 3B for details). We analyzed an iPSC line carrying a juvenile onset repeat (e.g., iPSC-C) to separate mutant HTT protein from the normal counterpart in immunoblot assays. Immunoblot analysis was performed using MAB2166 antibody (A; 4% gel; detecting both mutant and normal HTT protein) and mutant-specific 1F8 antibody (B; 6% gel; detecting polyQ protein). The upper band in EV-treated HD lines (middle lanes in panel A) and unique bands in EV-treated HD lines (middle lanes in panel B) represent full length mutant HTT protein (red arrows). A green arrow in panel A indicates normal HTT. Regions focusing on HTT protein are shown in Figure 3E and 3F. The 1F8 antibody was raised against polyglutamine tract, and therefore, smaller bands in panel B may represent non-specific and/or other polyglutamine-containing proteins. EV represents empty vector-treated control clones. NMD-CRISPR represents our mutant-specific NMD-CRISPR-Cas9 based on rs363099.

Supplemental Figure 8. The lack of fragmented mutant HTT protein in targeted clonal lines by our CRISPR-Cas9 strategy.

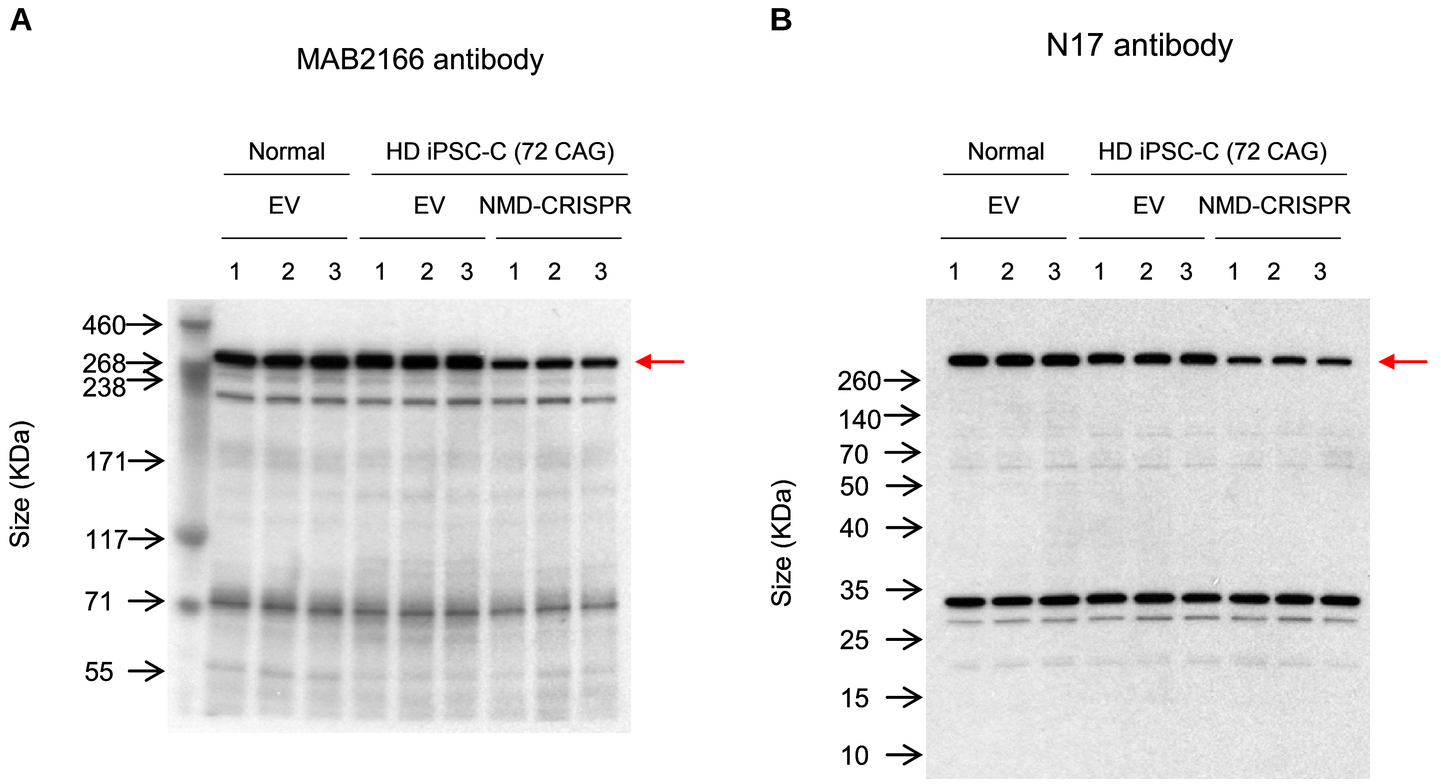

In order to determine whether our mutant-specific CRISPR-Cas9 strategy generates fragmented mutant HTT protein, we performed immunoblot analysis of non-HD controls and clonal lines established by empty-vector (EV) or NMD-CRISPR-Cas9 treatment (iPSC-C1, iPSC-C2, iPSC-C3). Focusing on evaluating smaller molecular weight proteins, whole cell extracts were resolved for a relatively short period of time (A, 6% gel for 55 min running; B, 4-20% gradient gel for 55 min running). Therefore, full length mutant and normal HTT proteins were not separated. Membranes were probed using MAB2166 antibody (A) and N-terminal antibody (B, N17). Red arrows indicate full length total HTT protein, representing the sum of mutant and normal HTT protein.

Supplemental Figure 9. Significant allele-specific reduction of mutant *HTT* mRNA.

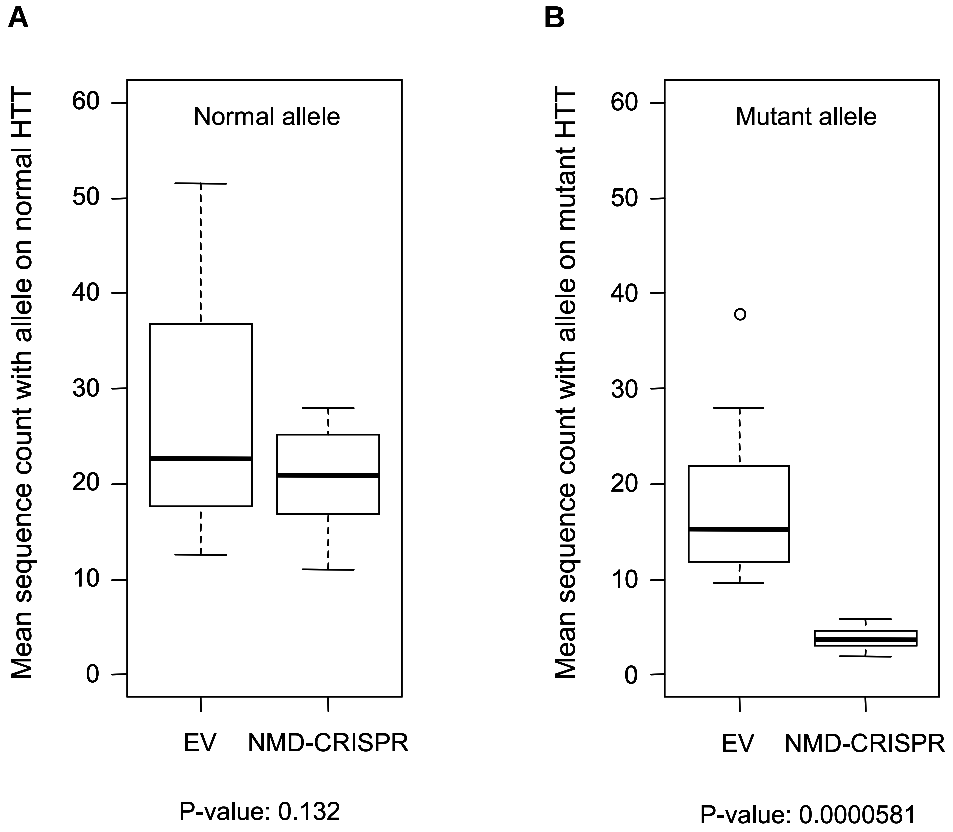

Ten sites are heterozygous in EV-treated (EV; n=12) and targeted clonal lines (CRISPR; n=12). For each heterozygous SNP site, allele-specific analysis (ASE) of RNAseq data generated 4 groups of read count data such as 1) number of counts of alleles on the normal *HTT* in EV-treated clones (A, EV), 2) counts of alleles on the normal *HTT* in CRISPR-Cas9-treated clones (A, NMD-CRISPR), 3) counts of alleles on the mutant *HTT* in EV-treated clones (B; EV), and 4) counts of alleles on the mutant *HTT* in CRISPR-Cas9-treated clones (B, NMD-CRISPR). We then calculated the mean read count for each group. Each box plot shows the distribution of read counts of 10 sites in 12 samples. P-values represent uncorrected nominal p-values from Student t-test (2-tailed; n=12). An uncorrected p-value less than 0.05 was considered nominally significant.

Supplemental Figure 10. Differential gene expression analysis comparing two groups of randomly assigned samples.

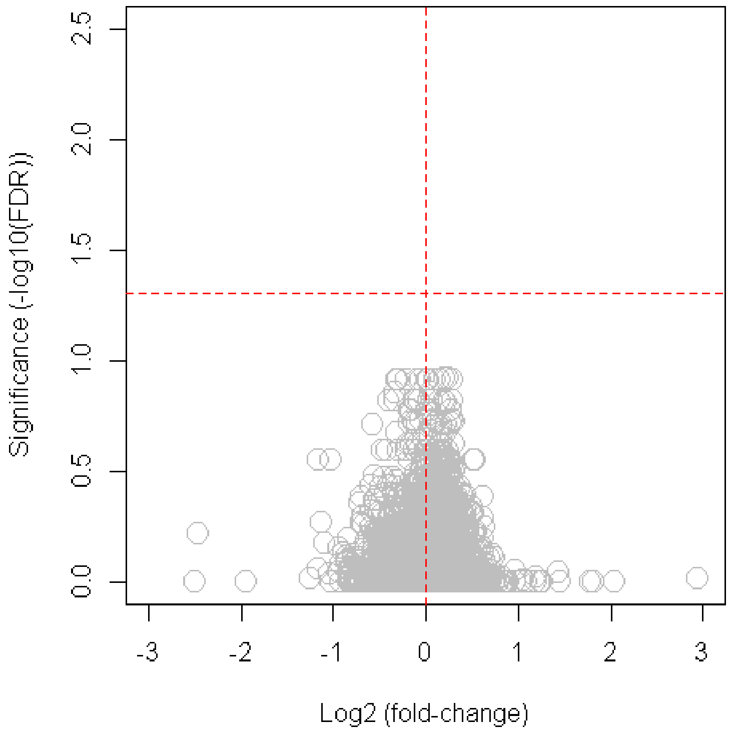

To explain the atypical shpe of the volcano plot in our DGE analysis of RNAseq data, we hypothesized that our RNAseq data represent a distribution of null statistics that do not involve significant differences except for *HTT.* To test this hypothesis, we performed DGE analysis comparing two groups of samples, each group comprising 12 randomly selected samples (6 EV-treated clones and 6 CRISPR-Cas9-treated clones). Subsequently, a volcano plot was drawn to evaluate the overall distribution of test statistics of random sample comparisons. Y-axis and X-axis represent significance (-log10(FDR)) and log2(fold-change), respectively. A red horizontal line represents the FDR significance (FDR, 0.05).

Supplemental Figure 11. GUIDE-seq assay to detect potential off-targets of our NMD-CRISPR-Cas9 strategy.

A

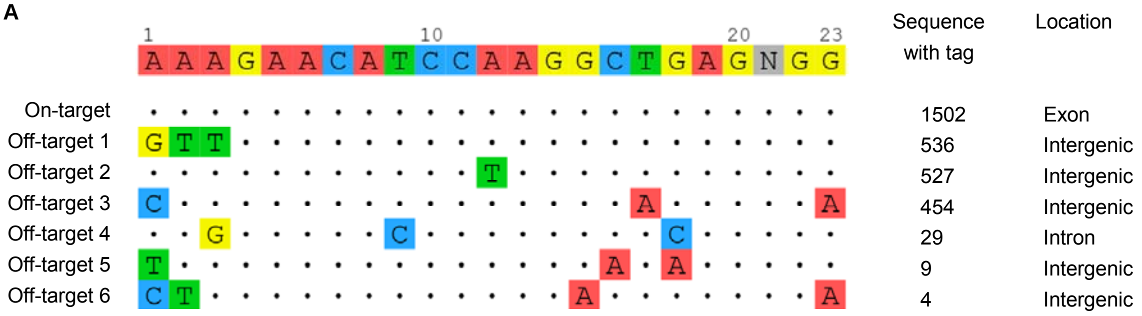

B

HD-derived cells	Site	Coordinate	% Editing	
			Empty vector	CRISPR-Cas9
iPSC-A (46 CAG) 2nd set	On-target	chr4:3162062	0	16.22
	Off-target 1	chr15:90327253	0	3.23
	Off-target 2	chr12:92165289	1.83	5.09
	Off-target 3	chr4:136067335	0	0.75
	Off-target 4	chr16:29994793	0	0
	Off-target 5	chr7: 152876948	0	0
	Off-target 6	chr12: 129528467	0	0
iPSC-B (42 CAG) 1st set	On-target	chr4:3162062	0	25.88
	Off-target 1	chr15:90327253	0	6.73
	Off-target 2	chr12:92165289	1.18	6.08
	Off-target 3	chr4:136067335	0	1.3
	Off-target 4	chr16: 29994793	0	0
	Off-target 5	chr7: 152876948	0	0
	Off-target 6	chr12: 129528467	0	0

A) The GUIDE-seq assay was performed to evaluate genome-wide off-target events in our NMD-CRISPR-Cas9 strategy. HEK-293T cells were treated with Cas9, our test gRNA, and a GUIDE-seq double-stranded oligodeoxynucleotide (dsODN) tag, followed by sequencing to identify dsODN integration as a proxy for CRISPR-Cas9-mediated double-strand breaks. The top line repessents the on-target site (*HTT*, 29th exon*)*; off-target locations with dsODN integration are shown below. Each dot and colored letter represent a perfect match and a mismatch, respectively. Number of sequence reads with tag represent the number of unique MiSeq sequencing reads containing dsODN, indicative of the levels of off-target editing.

B) Potential off-target sites identified in HEK293T cells were further evaluated in patient-derived iPSC lines. The second transfection experiment of iPSC-A and the first transfection experiment of iPSC-B which showed the highest editing efficiencies (refer to Supplemental Table 2) were subjected to PCR amplification using site-specific PCR primers and MiSeq analysis. Percent editing values represent the proportion of sequence reads

with indels out of all alleles for a given site. Corresponding empty vector-treated controls were also sequenced, showing low levels of modified alleles at off-target site #2 potentially due to sequencing errors.

Supplemental Figure 12. Allele specificity of NMD-CRISPR-Cas9 in differentiated neurons.

A

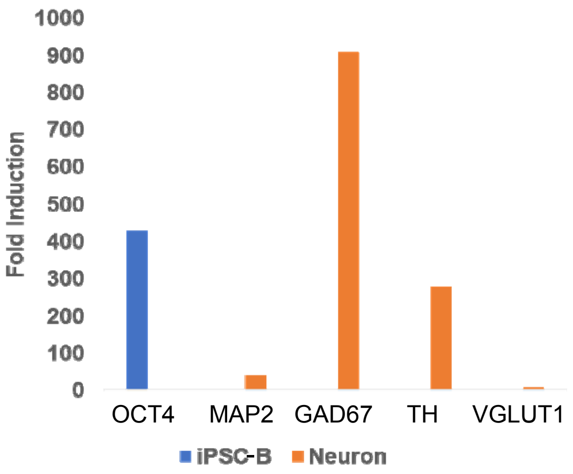

B

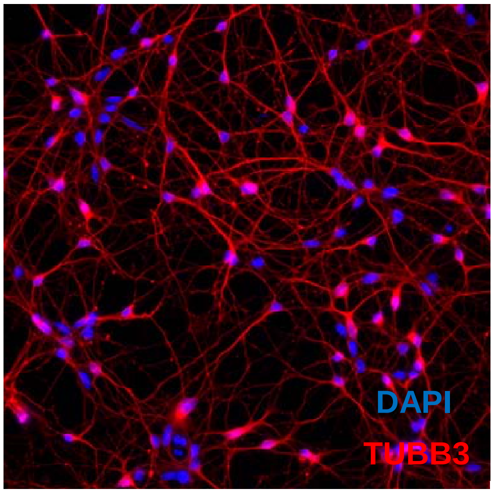

C

Allele	Description	Percentage of sequence reads	
		RNP delivery	mRNA delivery
Mutant *HTT*	Sequence reads with indels	4.2	2.5
	Sequence reads without indels	95.8	97.5
Normal *HTT*	Sequence reads with indels	0	0
	Sequence reads without indels	100	100

A) A patient-derived iPSC line with an adult onset CAG repeat (iPSC-B, 42 CAG) was differentiated into neurons. Subsequently, the expression levels of an iPSC marker (OCT4), neuronal markers (MAP2, GAD67, TH, VGLUT1), and a housekeeping gene (GAPDH) in iPSC-B (blue) and differentiated neurons (orange) were determined by qRT-PCR assays. Fold induction (Y-axis) of an iPSC marker and neuronal markers were calaulated relative to the expression levels of those in neurons and iPSC, respectively.

B) A representative neuronal marker staining is shown (magnification, 400X). DAPI (blue) and TUBB3 (red) represent nuclear and mature neurite marker, respectively.

C) Differentiated neurons were treated with RNP complex (RNP delivery; Cas9 protein-gRNA) or mRNA (mRNA delivery; Cas9 mRNA and gRNA), and then subjected to MiSeq analysis to evaluate the levels of editing efficiency and allele specificity.

Supplemental Table 1. A complete list of PAS on coding exons of *HTT.*

To develop allele-specific NMD-CRISPR-Cas9 strategies, PAS that generate or eliminate NGG PAM sites for wild-type *Sp*Cas9 on the coding sequence of *HTT* were identified by analyzing KGP (phase 3) data set. A total of 91 coding SNPs alter PAM sites for *Sp*Cas9 (i.e., NGG), representing candidate PAS that may permit allele-specific CRISPR-Cas9. Allele frequency (AF) was based on all populations in the KGP data set.

SNP ID	Position (hg19)	Reference	Alternative	AF (%)
rs184820567	3107140	C	T	0.019968
rs368339242	3107143	G	C	0.119808
rs567202171	3109053	G	C	0.019968
rs538952960	3109056	A	G	0.019968
rs374267492	3109063	C	T	0.019968
rs548352092	3117133	A	G	0.019968
rs181961779	3117891	C	T	0.019968
rs114476023	3122969	G	C	0.978435
rs371255782	3123015	C	T	0.019968
rs1065745	3123074	C	T	7.22843
rs374296062	3123101	C	T	0.019968
rs189018320	3123124	G	T	0.019968
rs545932099	3127303	C	G	0.019968
rs148032171	3129023	G	C	0.039936
rs536416239	3129043	C	T	0.019968
rs201861325	3129109	G	A	0.019968
rs2301367	3129124	G	A	0.039936
rs537369613	3129158	G	A	0.019968
rs573811022	3129205	C	T	0.019968
rs118005095	3129240	G	A	0.61901
rs576787839	3131750	G	A	0.019968
rs201938386	3132088	A	G	0.019968
rs374016128	3132131	C	T	0.019968
rs34389685	3133113	G	A	0.199681
rs150027738	3134406	T	C	0.079872
rs539073497	3136241	G	A	0.019968
rs192933099	3137663	A	C	0.019968
rs363075	3137674	G	A	2.3762
rs188101620	3138036	C	T	0.119808
rs549028339	3142283	C	A	0.019968
rs527729099	3146920	C	T	0.019968
rs1065746	3148624	G	C	0.019968
rs561719779	3148659	C	T	0.019968
rs1065747	3149780	C	G	0.019968
rs542613235	3149831	G	A	0.019968
rs200581635	3149844	C	T	0.019968
rs528411716	3149868	C	T	0.019968
rs188342053	3158882	C	A	0.019968
rs363099	3162056	C	T	21.3858
rs534902248	3174067	A	G	0.019968

rs534610900	3180138	G	A	0.019968
rs116293982	3182267	C	G	0.219649
rs150003356	3189510	T	C	0.09984
rs201878423	3201532	C	G	0.019968
rs558887183	3201637	C	G	0.019968
rs201094471	3208572	G	A	0.159744
rs200125263	3208575	C	G	0.019968
rs200077637	3208632	C	T	0.019968
rs540936636	3209018	C	T	0.039936
rs181217572	3209071	G	A	0.039936
rs201646519	3210567	T	C	0.09984
rs201104173	3210589	C	T	0.019968
rs547119123	3211676	G	T	0.019968
rs531260079	3213835	G	A	0.019968
rs565501560	3214317	C	T	0.019968
rs561152154	3215801	C	T	0.019968
rs573397591	3215822	C	T	0.139776
rs362331	3215835	T	C	44.1294
rs191063722	3216913	G	A	0.019968
rs377176195	3219553	G	A	0.019968
rs369228257	3224188	A	G	0.039936
rs182132217	3225168	C	T	0.179712
rs560138731	3225216	G	A	0.09984
rs570565498	3227431	C	T	0.019968
rs201944095	3230381	C	A	0.019968
rs201422922	3230431	C	G	0.039936
rs559860075	3230452	C	T	0.019968
rs547238016	3230668	C	T	0.019968
rs570616330	3230736	C	T	0.019968
rs536877956	3231619	A	G	0.019968
rs142891110	3231622	G	C	0.019968
rs573475907	3231643	G	C	0.019968
rs201467885	3231679	A	C	0.079872
rs200586785	3231706	A	G	0.019968
rs2229985	3231757	C	T	0.379393
rs529410176	3234904	G	A	0.039936
rs71608259	3235036	G	A	0.119808
rs144891713	3237100	C	T	0.019968
rs567166179	3237101	G	A	0.079872
rs573732436	3237314	G	T	0.019968
rs536481227	3237315	A	G	0.019968
rs199535935	3237376	C	T	0.119808
rs564865166	3237433	G	A	0.019968
rs200392606	3240187	T	C	0.019968
rs373377741	3240324	C	A	0.059904
rs371718523	3240685	C	G	0.019968
rs532740914	3240700	G	A	0.019968
rs563636870	3241573	C	T	0.019968
rs542631628	3241634	C	T	0.019968
rs200112468	3241720	C	T	0.039936
rs362308	3241735	T	C	0.059904

Supplemental Table 2. Allele specificities of rs363099-based NMD-CRISPR-Cas9.

Two independent iPSC lines with adult onset CAG repeats were co-transfected with Cas9 and our primary test gRNA depending on a PAM site generated by rs363099. Subsequently, bulk DNA samples were isolated without Puromycin selection, and subjected to PCR / MiSeq analysis. Three independent transfection experiments were performed for each line. $, percentage of out-of-frame indels; #, percentage of in-frame indels. MiSeq analysis of empty vector-treated patient-derived iPSC lines did not reveal any modification at the target site (not shown).

**A**	iPSC-A (46 CAG): Transfection without selection and MiSeq analysis			
Allele	Description	Number of sequence reads (%)		
		1st experiment	2nd experiment	3rd experiment
Mutant	Sequence with indel	1668 (16.03 $ / 0 #)	2390 (16.22 $ / 0 #)	2097 (15.97 $ / 0 #)
	Sequence w/o indel	8737 (83.97)	12345 (83.78)	11037 (84.03)
Normal	Sequence with indel	0 (0)	0 (0)	0 (0)
	Sequence w/o indel	11508 (100)	16111 (100)	13907 (100)

**B**	iPSC-B (42 CAG): Transfection without selection and MiSeq analysis			
Allele	Description	Number of sequence reads (%)		
		1st experiment	2nd experiment	3rd experiment
Mutant	Sequence with indel	4181 (24.98 $ / 0.9 #)	6421 (22.32 $ / 2.35 #)	6565 (22.45 $ / 1.12 #)
	Sequence w/o indel	11972 (74.12)	19607 (75.33)	21287 (76.43)
Normal	Sequence with indel	0 (0)	0 (0)	0 (0)
	Sequence w/o indel	19632 (100)	30898 (100)	32667 (100)

Supplemental Table 3. Characterization of targeted single cell clones for RNAseq analysis.

For RNAseq analysis, we transfected two iPSC lines carrying adult onset CAG repeats with PX551 EFS vector expressing *Sp*Cas9 and PX552 EFS vector expressing our primary test gRNA. Subsequently, a total of 12 independent single cell clones were established by limited dilution (without selection) for the experimental group. Twelve targeted clonal lines were then subjected to Sanger sequencing of genomic DNA, MiSeq analysis of genomic DNA, and cDNA to determine targeted alleles and expression levels of mutant and normal *HTT* mRNA, respectively. All targeted clones confirmed out-of-frame indels in the mutant *HTT* by Sanger sequencing and MiSeq of DNA; MiSeq analysis of cDNA confirmed those modifications. As observed in other clonal lines, targeted clonal lines for RNAseq analysis expressed a small amount of mutant *HTT* RNA (see cDNA MiSeq column), suggesting that MiSeq analysis detected newly produced mutant *HTT* mRNA that had not been subjected to NMD process. Percent values were relative to the sum of mutant and normal alleles.

Parental line	NMD-CRISPR-Cas9 Clone #	Allele	DNA Sanger	DNA MiSeq	cDNA MiSeq
			Modification	%	%
iPSC-A (46 CAG)	1	Mutant	1 nucleotide	50.57	8.4
		Normal	No change	49.43	91.6
	2	Mutant	2 nucleotides	54.09	7.0
		Normal	No change	45.91	93.0
	3	Mutant	1 nucleotide	51.12	8.8
		Normal	No change	48.88	91.2
	4	Mutant	5 nucleotides	48.33	9.1
		Normal	No change	51.67	90.9
	5	Mutant	1 nucleotide	50.88	9.5
		Normal	No change	49.12	90.5
	6	Mutant	5 nucleotides	47.26	8.2
		Normal	No change	52.74	91.8
iPSC-B (42 CAG)	1	Mutant	1 nucleotide	51.32	10.7
		Normal	No change	48.68	89.3
	2	Mutant	1 nucleotide	49.71	7.0
		Normal	No change	50.29	93.0
	3	Mutant	70 nucleotides	24.60	6.5
		Normal	No change	75.40	93.5
	4	Mutant	17 nucleotides	49.04	11.8
		Normal	No change	50.96	88.2
	5	Mutant	22 nucleotides	34.34	13.3
		Normal	No change	65.66	86.7
	6	Mutant	1 nucleotide	50.64	12.0
		Normal	No change	49.36	88.0

Supplemental Table 4. Empty vector treated control single cell clones for RNAseq analysis.

For control samples in RNAseq analysis, we also transfected same iPSC lines carrying adult onset CAG repeats with PX551 EFS vector expressing *Sp*Cas9 and empty PX552 EFS vector. Subsequently, a total of 12 independent clones were developed. All twelve control lines were subject to Sanger sequencing, confirming the lack of modification at the target site. For 6 selected clonal lines, we also performed MiSeq analysis of DNA, confirming the lack of modification and balanced allele counts in DNA. Further, we performed MiSeq analysis of cDNA samples for two selected clonal lines (iPSC-A1 and iPSC-B1), confirming the lack of modification and balanced expression of mutant and normal *HTT* in RNA. ND, not determined.

Parental line	Empty vector Clone #	Allele	DNA Sanger	DNA MiSeq	RNA MiSeq
			Modification	%	%
iPSC-A (46 CAG)	1	Mutant	None	49.1	48.5
		Normal	None	50.9	51.5
	2	Mutant	None	49.4	ND
		Normal	None	50.6	
	3	Mutant	None	49.4	ND
		Normal	None	50.6	
	4	Mutant	None	ND	ND
		Normal	None		
	5	Mutant	None	ND	ND
		Normal	None		
	6	Mutant	None	ND	ND
		Normal	None		
iPSC-B (42 CAG)	7	Mutant	None	49.5	49.1
		Normal	None	50.5	50.9
	8	Mutant	None	46.1	ND
		Normal	None	53.9	
	9	Mutant	None	47.1	ND
		Normal	None	52.9	
	10	Mutant	None	ND	ND
		Normal	None		
	11	Mutant	None	ND	ND
		Normal	None		
	12	Mutant	None	ND	ND
		Normal	None		

Supplemental Table 5. DGE analysis of RNAseq for potential off-targets identified by GUIDE-seq analysis.

GUIDE-seq analysis of HEK 293T cells revealed 6 potential off-target sites. To understand the impacts of our CRISPR-Cas9 strategy on the expression levels of potential off-target sites in patient-derived cells, we evaluated RNAseq data. Since 5 potential off-target sites are located in intergenic regions, we analyzed the nearest genes (genes in brackets). FDR represents adjusted p-value by false discovery rate.

GUIDE-seq analysis			RNAseq analysis		
Description	Chromosome	Position	Gene [nearest gene]	Fold-change	FDR
Off-target 1	5	90327260	[ANPEP]	0.97	0.9987
Off-target 2	2	92165307	[BTG1]	1.10	0.9987
Off-target 3	4	136067352	[PABPC4L]	0.96	0.9987
Off-target 4	16	29994793	TAOK2	1.03	0.9987
Off-target 5	7	152876948	[ACTR3B]	0.91	0.9987
Off-target 6	12	129528467	[TMEM132D]	1.08	0.9987

Supplemental Table 6. Predicted off-targets.

Potential off-target sites predicted by Cas-OFFinder website are summarized. Genomic coordinates were based on GRCh37/hg19. Genes in brackets represent the nearest genes to the predicted off-target site. Lower case letters in the sequence represent mismatches. Dashed lines represent bulges.

Chr	Coordinate	DNA	crRNA	Mismatch	Bulge size	Gene
1	94591387	AAAaAAC-TCCAAGGCTGAGTGG	AAAGAACATCCAAGGCTGAGNGG	1	1 (RNA)	[ABCA4]
1	94591387	AAA-AAacTCCAAGGCTGAGTGG	AAAGAACATCCAAGGCTGAGNGG	2	1 (RNA)	[ABCA4]
1	94591387	AAAaAACt-CCAAGGCTGAGTGG	AAAGAACATCCAAGGCTGAGNGG	2	1 (RNA)	[ABCA4]
1	94591387	AAAaAA-cTCCAAGGCTGAGTGG	AAAGAACATCCAAGGCTGAGNGG	2	1 (RNA)	[ABCA4]
1	227567892	AAAGAACATCtAAGAGCTGtGGGG	AAAGAACATCCAAG-GCTGAGNGG	2	1 (DNA)	[CDC42BPA]
2	26899611	tAAGAAg-TCCAAGGCTGAGGGG	AAAGAACATCCAAGGCTGAGNGG	2	1 (RNA)	[KCNK3]
2	26899611	tAAGAA-gTCCAAGGCTGAGGGG	AAAGAACATCCAAGGCTGAGNGG	2	1 (RNA)	[KCNK3]
2	60097499	AAAGAACAgtCAAGGCTGGAGAGG	AAAGAACATCCAAGGCT-GAGNGG	2	1 (DNA)	[MIR4432HG]
2	60097499	AAAGAACAgtCAAGGCTGGAGAGG	AAAGAACATCCAAGGCTG-AGNGG	2	1 (DNA)	[MIR4432HG]
2	64610397	AAAGAAC-TCCtAGGCaGAGGGG	AAAGAACATCCAAGGCTGAGNGG	2	1 (RNA)	[MIR4433B]
2	109069020	AAA-AACAaCCAAGGCaGAGAGG	AAAGAACATCCAAGGCTGAGNGG	2	1 (RNA)	GCC2
2	110450075	AAAcAAaATCCAA-GCTGAGAGG	AAAGAACATCCAAGGCTGAGNGG	2	1 (RNA)	[SOWAHC]
2	110450075	AAAcAAaATCCAAG-CTGAGAGG	AAAGAACATCCAAGGCTGAGNGG	2	1 (RNA)	[SOWAHC]
2	193426297	AAAGAACATCCAA-GaTGtGTGG	AAAGAACATCCAAGGCTGAGNGG	2	1 (RNA)	[PCGEM1]
2	193426297	AAAGAACATCCAAG-aTGtGTGG	AAAGAACATCCAAGGCTGAGNGG	2	1 (RNA)	[PCGEM1]
2	193426297	AAAGAACATCCAAGa-TGtGTGG	AAAGAACATCCAAGGCTGAGNGG	2	1 (RNA)	[PCGEM1]
2	218239665	AAAGAAttTCCAAGGCTGGAGAGG	AAAGAACATCCAAGGCT-GAGNGG	2	1 (DNA)	DIRC3
2	218239665	AAAGAAttTCCAAGGCTGGAGAGG	AAAGAACATCCAAGGCTG-AGNGG	2	1 (DNA)	DIRC3
2	227271147	AAAGAAgATaCAAGG-TGAGTGG	AAAGAACATCCAAGGCTGAGNGG	2	1 (RNA)	[LOC646736]
3	19663504	AAAG-ACATaCAAGGCTGAcTGG	AAAGAACATCCAAGGCTGAGNGG	2	1 (RNA)	[KCNH8]
3	19663504	AAAGA-CATaCAAGGCTGAcTGG	AAAGAACATCCAAGGCTGAGNGG	2	1 (RNA)	[KCNH8]
3	101648922	AAAGAAaATgCAAGGCT-AGGGG	AAAGAACATCCAAGGCTGAGNGG	2	1 (RNA)	[LINC02085]
3	121729858	AAAGAAtAgCCAA-GCTGAGAGG	AAAGAACATCCAAGGCTGAGNGG	2	1 (RNA)	ILDR1
3	121729858	AAAGAAtAgCCAAG-CTGAGAGG	AAAGAACATCCAAGGCTGAGNGG	2	1 (RNA)	ILDR1
3	126732945	AcAGAACATCCAgGG-TGAGTGG	AAAGAACATCCAAGGCTGAGNGG	2	1 (RNA)	PLXNA1
3	126732945	AcAGAACATCC-AGGgTGAGTGG	AAAGAACATCCAAGGCTGAGNGG	2	1 (RNA)	PLXNA1
3	126732945	AcAGAACATCCA-GGgTGAGTGG	AAAGAACATCCAAGGCTGAGNGG	2	1 (RNA)	PLXNA1
3	135757902	AAAGAACATCC-AGGCTGctGGG	AAAGAACATCCAAGGCTGAGNGG	2	1 (RNA)	PPP2R3A
3	135757902	AAAGAACATCCA-GGCTGctGGG	AAAGAACATCCAAGGCTGAGNGG	2	1 (RNA)	PPP2R3A
3	195922254	AAAGAA-ATCCcAGGCTGgGAGG	AAAGAACATCCAAGGCTGAGNGG	2	1 (RNA)	[ZDHHC19]
4	46524862	AAAaAACtTCC-AGGCTGAGTGG	AAAGAACATCCAAGGCTGAGNGG	2	1 (RNA)	[GABRA2]
4	46524862	AAAaAACtTCCA-GGCTGAGTGG	AAAGAACATCCAAGGCTGAGNGG	2	1 (RNA)	[GABRA2]

4	85525713	tAAGAAaA-CCAAGGCTGAGAGG	AAAGAACATCCAAGGCTGAGNGG	2	1 (RNA)	CDS1
4	85525713	tAAGAA-AaCCAAGGCTGAGAGG	AAAGAACATCCAAGGCTGAGNGG	2	1 (RNA)	CDS1
4	185093254	AAAGAgCATCCAgGGC-GAGAGG	AAAGAACATCCAAGGCTGAGNGG	2	1 (RNA)	ENPP6
4	185168971	AAAGAACcTaCAAGGCTG-GAGG	AAAGAACATCCAAGGCTGAGNGG	2	1 (RNA)	[ENPP6]
5	1868898	AAAGAA-ATtCAAGGCTtAGGGG	AAAGAACATCCAAGGCTGAGNGG	2	1 (RNA)	[IRX4]
5	11850226	AAAGtACAcCCAGAGGCTGAGGGG	AAAGAACATCCA-AGGCTGAGNGG	2	1 (DNA)	CTNND2
5	38446218	AgAGAAgATCCAA-GCTGAGCGG	AAAGAACATCCAAGGCTGAGNGG	2	1 (RNA)	EGFLAM
5	38446218	AgAGAAgATCCAAG-CTGAGCGG	AAAGAACATCCAAGGCTGAGNGG	2	1 (RNA)	EGFLAM
5	128388887	AAAGAACATCCAtGGaTTGAGAGG	AAAGAACATCCAAGGCT-GAGNGG	2	1 (DNA)	[SLC27A6]
5	128388887	AAAGAACATCCAtGGaTTGAGAGG	AAAGAACATCCAAGGC-TGAGNGG	2	1 (DNA)	[SLC27A6]
5	128388887	AAAGAACATCCAtGGAtTGAGAGG	AAAGAACATCCAAGG-CTGAGNGG	2	1 (DNA)	[SLC27A6]
5	138837396	AACAGAACATCtgAGGCTGAGGGG	AA-AGAACATCCAAGGCTGAGNGG	2	1 (DNA)	ECSCR
5	154300413	AAAGAA-AgaCAAGGCTGAGGGG	AAAGAACATCCAAGGCTGAGNGG	2	1 (RNA)	GEMIN5
5	157954509	AAAGAAg-TCCAAGGtTGAGGGG	AAAGAACATCCAAGGCTGAGNGG	2	1 (RNA)	[LOC101927697]
5	157954509	AAAGAA-gTCCAAGGtTGAGGGG	AAAGAACATCCAAGGCTGAGNGG	2	1 (RNA)	[LOC101927697]
6	65842806	cAAGAACAgCCAA-GCTGAGAGG	AAAGAACATCCAAGGCTGAGNGG	2	1 (RNA)	EYS
6	65842806	cAAGAACAgCCAAG-CTGAGAGG	AAAGAACATCCAAGGCTGAGNGG	2	1 (RNA)	EYS
7	57487310	AtgGAACA-CCAAGGCTGAGAGG	AAAGAACATCCAAGGCTGAGNGG	2	1 (RNA)	LOC100653233
7	63645222	AtgGAACA-CCAAGGCTGAGAGG	AAAGAACATCCAAGGCTGAGNGG	2	1 (RNA)	[ZNF735]
7	120098678	AAAAGAgaATCCAAGGCTGAGAGG	A-AAGAACATCCAAGGCTGAGNGG	2	1 (DNA)	KCND2
7	120098678	AAAAGAgaATCCAAGGCTGAGAGG	AAA-GAACATCCAAGGCTGAGNGG	2	1 (DNA)	KCND2
7	120098678	AAAAGAgaATCCAAGGCTGAGAGG	AA-AGAACATCCAAGGCTGAGNGG	2	1 (DNA)	KCND2
7	120098679	AAAGAgaATCCAAGGCTGAGAGG	AAAGAACATCCAAGGCTGAGNGG	2	0 (X)	KCND2
7	120098680	A-AGAgaATCCAAGGCTGAGAGG	AAAGAACATCCAAGGCTGAGNGG	2	1 (RNA)	KCND2
7	120098680	AA-GAgaATCCAAGGCTGAGAGG	AAAGAACATCCAAGGCTGAGNGG	2	1 (RNA)	KCND2
7	149550143	AAAGAAgAaCCAAGG-TGAGGGG	AAAGAACATCCAAGGCTGAGNGG	2	1 (RNA)	ZNF862
7	152876931	A-AGAACATCCAAGGaTaAGTGG	AAAGAACATCCAAGGCTGAGNGG	2	1 (RNA)	[LINC01287]
7	152876931	AA-GAACATCCAAGGaTaAGTGG	AAAGAACATCCAAGGCTGAGNGG	2	1 (RNA)	[LINC01287]
8	41177158	AAAGcAC-TCCAAGGCTaAGTGG	AAAGAACATCCAAGGCTGAGNGG	2	1 (RNA)	[SFRP1]
9	12181344	AAAGAACAT-CAAGGaTtAGAGG	AAAGAACATCCAAGGCTGAGNGG	2	1 (RNA)	[TYRP1]
9	12181344	AAAGAACATC-AAGGaTtAGAGG	AAAGAACATCCAAGGCTGAGNGG	2	1 (RNA)	[TYRP1]
9	39050426	cAAcAACATCCAAGG-TGAGAGG	AAAGAACATCCAAGGCTGAGNGG	2	1 (RNA)	[CNTNAP3]
9	39050449	cAAcAACATCCAAGG-TGAGAGG	AAAGAACATCCAAGGCTGAGNGG	2	1 (RNA)	[CNTNAP3]
9	65685470	cAAcAACATCCAAGG-TGAGAGG	AAAGAACATCCAAGGCTGAGNGG	2	1 (RNA)	[CNTNAP3P2]
9	116590974	AAAGAAaA-gCAAGGCTGAGTGG	AAAGAACATCCAAGGCTGAGNGG	2	1 (RNA)	[ZNF618]
9	116590974	AAAGAAaAg-CAAGGCTGAGTGG	AAAGAACATCCAAGGCTGAGNGG	2	1 (RNA)	[ZNF618]
9	116590974	AAAGAAaAgC-AAGGCTGAGTGG	AAAGAACATCCAAGGCTGAGNGG	2	1 (RNA)	[ZNF618]
9	116590974	AAAGAA-AagCAAGGCTGAGTGG	AAAGAACATCCAAGGCTGAGNGG	2	1 (RNA)	[ZNF618]
9	133907798	AAgGAACcTCC-AGGCTGAGGGG	AAAGAACATCCAAGGCTGAGNGG	2	1 (RNA)	LAMC3
9	133907798	AAgGAACcTCCA-GGCTGAGGGG	AAAGAACATCCAAGGCTGAGNGG	2	1 (RNA)	LAMC3

10	13608321	AAAGAACccCCAAGGCTG-GGGG	AAAGAACATCCAAGGCTGAGNGG	2	1 (RNA)	[PRPF18]
10	61614633	AAAGAACA-gaAAGGCTGAGAGG	AAAGAACATCCAAGGCTGAGNGG	2	1 (RNA)	CCDC6
10	61614633	AAAGAACAg-aAAGGCTGAGAGG	AAAGAACATCCAAGGCTGAGNGG	2	1 (RNA)	CCDC6
10	61614633	AAAGAACAga-AAGGCTGAGAGG	AAAGAACATCCAAGGCTGAGNGG	2	1 (RNA)	CCDC6
10	115830553	AAAGAACtaCCAAGGCTG-GCGG	AAAGAACATCCAAGGCTGAGNGG	2	1 (RNA)	[ADRB1]
11	17899475	AAAGAAC-TCacAGGCTGAGGGG	AAAGAACATCCAAGGCTGAGNGG	2	1 (RNA)	SERGEF
11	27318534	tAAGAAC-TCCAAGGtTGAGGGG	AAAGAACATCCAAGGCTGAGNGG	2	1 (RNA)	[CCDC34]
11	27407987	AAAGAAg-TCCAAGGCTGtGAGG	AAAGAACATCCAAGGCTGAGNGG	2	1 (RNA)	LGR4
11	27407987	AAAGAA-gTCCAAGGCTGtGAGG	AAAGAACATCCAAGGCTGAGNGG	2	1 (RNA)	LGR4
11	37981053	cAAtAACATCC-AGGCTGAGAGG	AAAGAACATCCAAGGCTGAGNGG	2	1 (RNA)	[LOC105376633]
11	37981053	cAAtAACATCCA-GGCTGAGAGG	AAAGAACATCCAAGGCTGAGNGG	2	1 (RNA)	[LOC105376633]
11	39246550	AAAGAACAcCCAAGGCaG-GAGG	AAAGAACATCCAAGGCTGAGNGG	2	1 (RNA)	[LINC01493]
11	69708486	AAAGAGACccCCAAGGCTGAGAGG	AAAGA-ACATCCAAGGCTGAGNGG	2	1 (DNA)	[FGF3]
11	89301552	cAAcAACATCCAA-GCTGAGAGG	AAAGAACATCCAAGGCTGAGNGG	2	1 (RNA)	NOX4
11	89301552	cAAcAACATCCAAG-CTGAGAGG	AAAGAACATCCAAGGCTGAGNGG	2	1 (RNA)	NOX4
11	131203198	AAgGAACA-gCAAGGCTGAGAGG	AAAGAACATCCAAGGCTGAGNGG	2	1 (RNA)	[NTM]
11	131203198	AAgGAACAg-CAAGGCTGAGAGG	AAAGAACATCCAAGGCTGAGNGG	2	1 (RNA)	[NTM]
11	131203198	AAgGAACAgC-AAGGCTGAGAGG	AAAGAACATCCAAGGCTGAGNGG	2	1 (RNA)	[NTM]
12	92165289	gAAAGAACATCCtAGGCTGAGTGG	A-AAGAACATCCAAGGCTGAGNGG	2	1 (DNA)	[LINC01619]
12	92165289	gAAAGAACATCCtAGGCTGAGTGG	AAA-GAACATCCAAGGCTGAGNGG	2	1 (DNA)	[LINC01619]
12	92165289	gAAAGAACATCCtAGGCTGAGTGG	AA-AGAACATCCAAGGCTGAGNGG	2	1 (DNA)	[LINC01619]
12	92165290	AAAGAACATCCtAGGCTGAGTGG	AAAGAACATCCAAGGCTGAGNGG	1	0 (X)	[LINC01619]
12	92165291	A-AGAACATCCtAGGCTGAGTGG	AAAGAACATCCAAGGCTGAGNGG	1	1 (RNA)	[LINC01619]
12	92165291	AA-GAACATCCtAGGCTGAGTGG	AAAGAACATCCAAGGCTGAGNGG	1	1 (RNA)	[LINC01619]
12	92165291	AAg-AACATCCtAGGCTGAGTGG	AAAGAACATCCAAGGCTGAGNGG	2	1 (RNA)	[LINC01619]
12	99282433	AAAGAACAcCCcAGGCT-AGGGG	AAAGAACATCCAAGGCTGAGNGG	2	1 (RNA)	ANKS1B
12	120161757	AAAGAACATCCAAaGCTTGAGGGG	AAAGAACATCCAAGGCT-GAGNGG	1	1 (DNA)	CIT
12	120161757	AAAGAACATCCAAaGCTTGAGGGG	AAAGAACATCCAAGGC-TGAGNGG	1	1 (DNA)	CIT
12	120161757	AAAGAACATCCAAaGCTt-GAGG	AAAGAACATCCAAGGCTGAGNGG	2	1 (RNA)	CIT
12	120161757	AAAGAACATCCAAAGctTGAGGGG	AAAGAACATCCA-AGGCTGAGNGG	2	1 (DNA)	CIT
12	120161757	AAAGAACATCCAAAGctTGAGGGG	AAAGAACATCC-AAGGCTGAGNGG	2	1 (DNA)	CIT
12	120161757	AAAGAACATCCAAaGCT-tGAGG	AAAGAACATCCAAGGCTGAGNGG	2	1 (RNA)	CIT
12	120161757	AAAGAACATCCAAAGctTGAGGGG	AAAGAACATCCAA-GGCTGAGNGG	2	1 (DNA)	CIT
12	120161757	AAAGAACATCCAAaGCtTGAGGGG	AAAGAACATCCAAGG-CTGAGNGG	2	1 (DNA)	CIT
12	120161757	AAAGAACATCCAAaGCTtGAGGGG	AAAGAACATCCAAGGCTG-AGNGG	2	1 (DNA)	CIT
13	73321278	AAAGAACA-CCAAGGCaGtGAGG	AAAGAACATCCAAGGCTGAGNGG	2	1 (RNA)	BORA
14	49144352	cAAtAACATCC-AGGCTGAGAGG	AAAGAACATCCAAGGCTGAGNGG	2	1 (RNA)	[LINC00648]
14	49144352	cAAtAACATCCA-GGCTGAGAGG	AAAGAACATCCAAGGCTGAGNGG	2	1 (RNA)	[LINC00648]
14	65035930	AAAGgtCA-CCAAGGCTGAGAGG	AAAGAACATCCAAGGCTGAGNGG	2	1 (RNA)	PPP1R36
14	102426650	AAAGAAaATCCAAGGCT-AGAGG	AAAGAACATCCAAGGCTGAGNGG	1	1 (RNA)	[DYNC1H1]

14	102426650	AAAGAAaATCCAAGGCTa-GAGG	AAAGAACATCCAAGGCTGAGNGG	2	1 (RNA)	[DYNC1H1]
14	102426650	AAAGAAaATCCAAGGC-tAGAGG	AAAGAACATCCAAGGCTGAGNGG	2	1 (RNA)	[DYNC1H1]
15	60445586	tAAGAAaA-CCAAGGCTGAGAGG	AAAGAACATCCAAGGCTGAGNGG	2	1 (RNA)	[FOXB1]
15	60445586	tAAGAA-AaCCAAGGCTGAGAGG	AAAGAACATCCAAGGCTGAGNGG	2	1 (RNA)	[FOXB1]
15	84243595	AAAGcACATCCgA-GCTGAGGGG	AAAGAACATCCAAGGCTGAGNGG	2	1 (RNA)	SH3GL3
15	84243595	AAAGcACATCCgAG-CTGAGGGG	AAAGAACATCCAAGGCTGAGNGG	2	1 (RNA)	SH3GL3
15	90327253	t-tGAACATCCAAGGCTGAGAGG	AAAGAACATCCAAGGCTGAGNGG	2	1 (RNA)	[ANPEP]
15	90327253	tt-GAACATCCAAGGCTGAGAGG	AAAGAACATCCAAGGCTGAGNGG	2	1 (RNA)	[ANPEP]
15	90327253	AGttGAACATCCAAGGCTGAGAGG	A-AAGAACATCCAAGGCTGAGNGG	2	1 (DNA)	[ANPEP]
15	90327253	AgtTGAACATCCAAGGCTGAGAGG	AAA-GAACATCCAAGGCTGAGNGG	2	1 (DNA)	[ANPEP]
15	90327253	AgTtGAACATCCAAGGCTGAGAGG	AA-AGAACATCCAAGGCTGAGNGG	2	1 (DNA)	[ANPEP]
16	1905010	AAAGgACAgCCAGAGGCTGAGAGG	AAAGAACATCCA-AGGCTGAGNGG	2	1 (DNA)	MEIOB
16	14500236	A-AGAACATCacAGGCTGAGGGG	AAAGAACATCCAAGGCTGAGNGG	2	1 (RNA)	[PARN]
16	14500236	AA-GAACATCacAGGCTGAGGGG	AAAGAACATCCAAGGCTGAGNGG	2	1 (RNA)	[PARN]
16	14500236	AGAAGAACATCacAGGCTGAGGGG	A-AAGAACATCCAAGGCTGAGNGG	2	1 (DNA)	[PARN]
16	14604020	AAAGAACAgCaAAGGCTG-GGGG	AAAGAACATCCAAGGCTGAGNGG	2	1 (RNA)	PARN
16	24820222	AAGAGAAtATCCAAGtCTGAGGGG	AA-AGAACATCCAAGGCTGAGNGG	2	1 (DNA)	TNRC6A
16	27769986	A-AGAACATtCcAGGCTGAGAGG	AAAGAACATCCAAGGCTGAGNGG	2	1 (RNA)	KIAA0556
16	27769986	AA-GAACATtCcAGGCTGAGAGG	AAAGAACATCCAAGGCTGAGNGG	2	1 (RNA)	KIAA0556
16	27769986	AGAAGAACATtCcAGGCTGAGAGG	A-AAGAACATCCAAGGCTGAGNGG	2	1 (DNA)	KIAA0556
16	53553406	AAgGAACATCC-AGGCaGAGCGG	AAAGAACATCCAAGGCTGAGNGG	2	1 (RNA)	[AKTIP]
16	53553406	AAgGAACATCCA-GGCaGAGCGG	AAAGAACATCCAAGGCTGAGNGG	2	1 (RNA)	[AKTIP]
16	73051152	AAAG-AtATCaAAGGCTGAGTGG	AAAGAACATCCAAGGCTGAGNGG	2	1 (RNA)	ZFHX3
16	73051152	AAAGA-tATCaAAGGCTGAGTGG	AAAGAACATCCAAGGCTGAGNGG	2	1 (RNA)	ZFHX3
16	73051152	AAAGAt-ATCaAAGGCTGAGTGG	AAAGAACATCCAAGGCTGAGNGG	2	1 (RNA)	ZFHX3
17	10639941	AAAcAAtATCCAAGG-TGAGTGG	AAAGAACATCCAAGGCTGAGNGG	2	1 (RNA)	TMEM220-AS1
17	63425587	gAAGAAC-TCCAAGGgTGAGTGG	AAAGAACATCCAAGGCTGAGNGG	2	1 (RNA)	[CRAT40]
18	29136616	AAAGAAaATGCCAAGGCTGAtTGG	AAAGAACAT-CCAAGGCTGAGNGG	2	1 (DNA)	DSG2-AS1
18	32182544	A-AGAACcTCtAAGGCTGAGTGG	AAAGAACATCCAAGGCTGAGNGG	2	1 (RNA)	DTNA
18	32182544	AA-GAACcTCtAAGGCTGAGTGG	AAAGAACATCCAAGGCTGAGNGG	2	1 (RNA)	DTNA
18	45913442	AAAGAACATtC-AGGtTGAGGGG	AAAGAACATCCAAGGCTGAGNGG	2	1 (RNA)	ZBTB7C
18	45913442	AAAGAACATtCA-GGtTGAGGGG	AAAGAACATCCAAGGCTGAGNGG	2	1 (RNA)	ZBTB7C
19	767169	AAAGAAaAT-CAtGGCTGAGAGG	AAAGAACATCCAAGGCTGAGNGG	2	1 (RNA)	[MISP]
19	767169	AAAGAAaATC-AtGGCTGAGAGG	AAAGAACATCCAAGGCTGAGNGG	2	1 (RNA)	[MISP]
19	7291791	AAAGAAaATCCAA-GaTGAGTGG	AAAGAACATCCAAGGCTGAGNGG	2	1 (RNA)	INSR
19	7291791	AAAGAAaATCCAAG-aTGAGTGG	AAAGAACATCCAAGGCTGAGNGG	2	1 (RNA)	INSR
19	7291791	AAAGAAaATCCAAGa-TGAGTGG	AAAGAACATCCAAGGCTGAGNGG	2	1 (RNA)	INSR
19	35328785	AAAGAACAggC-AGGCTGAGTGG	AAAGAACATCCAAGGCTGAGNGG	2	1 (RNA)	[LINC01801]
19	35328785	AAAGAACAggCA-GGCTGAGTGG	AAAGAACATCCAAGGCTGAGNGG	2	1 (RNA)	[LINC01801]
23	65573412	AAA-AAaATCCcAGGCTGAGAGG	AAAGAACATCCAAGGCTGAGNGG	2	1 (RNA)	[HEPH]

23	65573412	AAAaAA-ATCCcAGGCTGAGAGG	AAAGAACATCCAAGGCTGAGNGG	2	1 (RNA)	[HEPH]
23	78841448	tAAcAACATCCAA-GCTGAGAGG	AAAGAACATCCAAGGCTGAGNGG	2	1 (RNA)	[ITM2A]
23	78841448	tAAcAACATCCAAG-CTGAGAGG	AAAGAACATCCAAGGCTGAGNGG	2	1 (RNA)	[ITM2A]
23	147745920	AAAG-ACATtCtAGGCTGAGGGG	AAAGAACATCCAAGGCTGAGNGG	2	1 (RNA)	AFF2
23	147745920	AAAGA-CATtCtAGGCTGAGGGG	AAAGAACATCCAAGGCTGAGNGG	2	1 (RNA)	AFF2

Supplemental Table 7. MiSeq analysis of pooled genomic DNA for the best predicted off-target.

GUIDE-seq analysis and subsequent genomic annotation did not predict significant impacts of rs363099-based CRISPR-Cas9 strategy on the expression levels of protein-coding genes because potential off-target sites reside at intergenic regions or introns. In addition, RNAseq data analysis of targeted single cell clones did not reveal recurrent off-targeting. We further evaluated algorithmically predicted off-target sites (refer to Supplemental Table 6 for full list), focusing on the best predicted off-target site (chr12: 92165291-92165313 with one mismatch). For this, we transfected iPSC-C cells with *Sp*Cas9 and our test gRNA, and performed MiSeq analysis of pooled DNA sample. A total of 112,514 MiSeq sequencing reads were analyzed (n=1), but none of them involved indels, indicating this site was not targeted.

Locus	Type	Number of reads (%)
chr12: 92165291-92165313	Sequence w/o indels	112,514 (100)
	Sequence with indels	0 (0)

Supplemental Table 8. Sequences of oligos for cloning and PCR.

	Name	Sequence	Description
Oligo	Oligo 1	caccgAAAGAACATCCAAGGCTGAG	Cloning of gRNA into lentiCRISPRv2 vecto
	Oligo 2	aaacCTCAGCCTTGGATGTTCTTTc	
	Oligo 1	accAAAGAACATCCAAGGCTGAG	Cloning of gRNA into PX552 vector
	Oligo 2	aacCTCAGCCTTGGATGTTCTTT	
Primer	Forward 1	CCAGTAACCGTGTGTTCTC	DNA and RNA Miseq
	Reverse 1	CCTAGATGAACTCAGCCCA	
	Forward 2	ATGAAGGCCTTCGAGTCCC	*HTT* CAG region PCR
	Reverse 2	GGCTGAGGAAGCTGAGGA	
	Forward 3	GAAAGCTAAAGATGTAGAGTCAGCA	MiSeq analysis of the best predicted off-target
	Reverse 3	TCATGCTTAGGCTCTGATTCC	
	Forward 4	CTGCTGGACTCCATGATTC	Miseq analysis of off-target #1
	Reverse 4	TGGTGAGCATTTCTTGACAAATG	
	Forward 5	TAGAGTCAGCAGAGATAGGGA	Miseq analysis of off-target #2
	Reverse 5	TCATGCTTAGGCTCTGATTC	
	Forward 6	TAGTGGCAAGAAATAGAGAACATTTGT	Miseq analysis of off-target #3
	Reverse 6	GTGTGTTCCAGCACTCTG	
	Forward 7	GTACACAGCTCACCCTTG	Miseq analysis of off-target #4
	Reverse 7	GGTTATCTCTGGCTGGTAGG	
	Forward 8	GCATTCAACAGAGGCTTCC	Miseq analysis of off-target #5
	Reverse 8	GTTGTGCTGTTTTAGAGCTCAT	
	Forward 9	AGATCAATTTATGGTATCTTCTCTCTCTCT	Miseq analysis of off-target #6
	Reverse 9	CCTTCGAAGGTAGACTCCA	

Supplemental Table 9. Oligos and primers for GIDE-seq assays.

Oligos required for next-generation sequencing library prep PCR #1

oligo ID	sequence	description
oBK8995	GTGTGTTCTCTCCTTCACCTTCCC	P5 primer for HTT exon 29 SpCas9 target site
oBK8996	GCACCCTGGAGAACCAGGC	P7 primer for HTT exon 29 SpCas9 target site

Oligos required for next-generation sequencing library prep PCR #2

oligo ID	description	i5	i5 barcode	sequence
oRW1116	Nextera DNA index 501	A501	TAGATCGC	AATGATACGGCGACCACCGAGATCTACACTAGATCGCACACTCTTTCCCTACACGACGCTCTTCCGATCT
oRW1118	Nextera DNA index 503	A503	TATCCTCT	AATGATACGGCGACCACCGAGATCTACACTATCCTCTACACTCTTTCCCTACACGACGCTCTTCCGATCT
oRW1120	Nextera DNA index 505	A505	GTAAGGAG	AATGATACGGCGACCACCGAGATCTACACGTAAGGAGACACTCTTTCCCTACACGACGCTCTTCCGATCT
oRW1092	Nextera DNA index 701	A701	TCGCCTTA	CAAGCAGAAGACGGCATACGAGATTCGCCTTAGTGACTGGAGTTCAGACGTGTGCTCTTCCGATCT
oRW1093	Nextera DNA index 702	A702	CTAGTACG	CAAGCAGAAGACGGCATACGAGATCTAGTACGGTGACTGGAGTTCAGACGTGTGCTCTTCCGATCT
oRW1094	Nextera DNA index 703	A703	TTCTGCCT	CAAGCAGAAGACGGCATACGAGATTTCTGCCTGTGACTGGAGTTCAGACGTGTGCTCTTCCGATCT
oRW1098	Nextera DNA index 707	A707	GTAGAGAG	CAAGCAGAAGACGGCATACGAGATGTAGAGAGGTGACTGGAGTTCAGACGTGTGCTCTTCCGATCT
oRW1099	Nextera DNA index 708	A708	CCTCTCTG	CAAGCAGAAGACGGCATACGAGATCCTCTCTGGTGACTGGAGTTCAGACGTGTGCTCTTCCGATCT
oRW1100	Nextera DNA index 709	A709	AGCGTAGC	CAAGCAGAAGACGGCATACGAGATAGCGTAGCGTGACTGGAGTTCAGACGTGTGCTCTTCCGATCT

Oligos required for GUIDE-seq library sequencing

oligo ID	sequence
Index1	ATCACCGACTGCCCATAGAGAGGACTCCAGTCAC
Read2	GTGACTGGAGTCCTCTCTATGGGCAGTCGGTGAT

Sample information for GUIDE-seq libraries

Sample Description	P7 Sequence (Barcode 1)	Adapter Sequence (Barcode 2)	SpCas9 target site sequence with PAM
HEK 293T cells - gRNA targeting *HTT* exon 29	TAAGGCGA	TAGATCGC	AAAGAACATCCAAGGCTGAGCGG
HEK 293T cells - no gRNA, dsODN control	CGTACTAG	CTCTCTAT	n/a

Supplemental References

1. Dobin, A., Davis, C.A., Schlesinger, F., Drenkow, J., Zaleski, C., Jha, S., Batut, P., Chaisson, M., and Gingeras, T.R. 2013. STAR: ultrafast universal RNA-seq aligner. *Bioinformatics* 29:15-21.
2. Lee, J.M., Kim, K.H., Shin, A., Chao, M.J., Abu Elneel, K., Gillis, T., Mysore, J.S., Kaye, J.A., Zahed, H., Kratter, I.H., et al. 2015. Sequence-Level Analysis of the Major European Huntington Disease Haplotype. *Am J Hum Genet* 97:435-444.
3. Vera Alvarez, R., Pongor, L.S., Marino-Ramirez, L., and Landsman, D. 2019. TPMCalculator: one-step software to quantify mRNA abundance of genomic features. *Bioinformatics* 35:1960-1962.
4. Tsai, S.Q., Zheng, Z., Nguyen, N.T., Liebers, M., Topkar, V.V., Thapar, V., Wyvekens, N., Khayter, C., Iafrate, A.J., Le, L.P., et al. 2015. GUIDE-seq enables genome-wide profiling of off-target cleavage by CRISPR-Cas nucleases. *Nat Biotechnol* 33:187-197.
5. Kleinstiver, B.P., Sousa, A.A., Walton, R.T., Tak, Y.E., Hsu, J.Y., Clement, K., Welch, M.M., Horng, J.E., Malagon-Lopez, J., Scarfo, I., et al. 2019. Engineered CRISPR-Cas12a variants with increased activities and improved targeting ranges for gene, epigenetic and base editing. *Nat Biotechnol* 37:276-282.
6. Schenkel, L.C., Kerkhof, J., Stuart, A., Reilly, J., Eng, B., Woodside, C., Levstik, A., Howlett, C.J., Rupar, A.C., Knoll, J.H.M., et al. 2016. Clinical Next-Generation Sequencing Pipeline Outperforms a Combined Approach Using Sanger Sequencing and Multiplex Ligation-Dependent Probe Amplification in Targeted Gene Panel Analysis. *J Mol Diagn* 18:657-667.
7. Clement, K., Rees, H., Canver, M.C., Gehrke, J.M., Farouni, R., Hsu, J.Y., Cole, M.A., Liu, D.R., Joung, J.K., Bauer, D.E., et al. 2019. CRISPResso2 provides accurate and rapid genome editing sequence analysis. *Nat Biotechnol* 37:224-226.
8. Fjodorova, M., and Li, M. 2018. Robust Induction of DARPP32-Expressing GABAergic Striatal Neurons from Human Pluripotent Stem Cells. *Methods Mol Biol* 1780:585-605.
